# Multi‐Dimensional Physical‐Layer Security via Digitally Mediated Space–Time Modulation

**DOI:** 10.1002/advs.77688

**Published:** 2026-09-27

**Authors:** Kejin Chen, Heng Mao, Feng Yang, Gengbo Wu, Lei Zhang, Yikai Chen, Shi‐Wei Qu, Jun Hu, Shiwen Yang

**Affiliations:** ^1^ School of Electronic Science and Engineering University of Electronic Science and Technology of China (UESTC) Chengdu China; ^2^ State Key Laboratory of Terahertz and Millimeter Waves, Department of Electrical Engineering City University of Hong Kong Hong Kong China; ^3^ State Key Laboratory of Millimeter Waves, School of Information Science and Engineering Southeast University Nanjing China

**Keywords:** communications system, degrees of freedom, electronic engineering, modulation, observability, secure transmission

## Abstract

Modern sensing and communication systems increasingly operate in congested electromagnetic environments, where suppressing the observability of radiated signals at unintended receivers is essential for secure operation. Space–time modulation offers a powerful means of shaping radiation for secure transmission by exploiting temporal degrees of freedom, but its achievable control is often limited by hardware‐bound modulation dimensions, with further expansion incurring substantial system complexity. Here, we propose a new digitally mediated space–time modulation paradigm that significantly expands multi‐dimensional radiation‐control capability with minimal hardware overhead. By coherently coupling digital baseband processing with analog time modulation, the proposed architecture enables simultaneous multi‐beam transmission of distinct waveforms while effectively reducing waveform observability in unintended spatial regions. Numerical simulations and experimental measurements of a multi‐target prototype verify that complex radiation behaviours and intrinsic physical‐layer security can be achieved using simple periodic modulation, providing a versatile strategy for radiation‐domain control in next‐generation sensing and communication systems.

## Graphical abstract

Digitally mediated space–time modulation moves radiation control beyond hardware‐bound switching. Multiple information‐bearing and masking waveforms are jointly synthesized at baseband and coherently coupled to simple periodic time modulation, enabling programmable, independently directed multi‐beam radiation without increasing switching‐state complexity. This architecture expands radiation‐control degrees of freedom while preserving low‐complexity hardware.

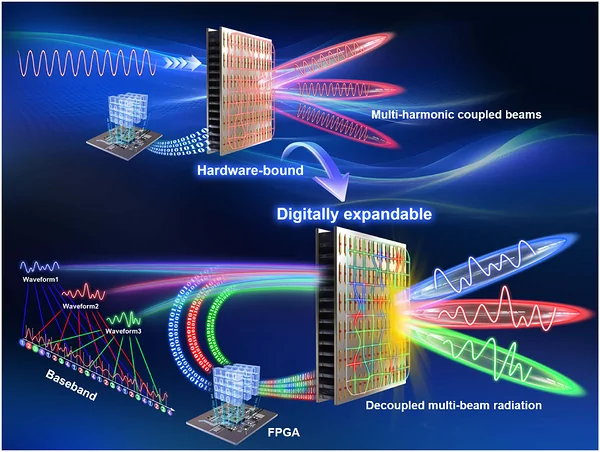

## 1 Introduction

Time varying property is one of the most ubiquitous characteristics of natural phenomena. However, in electromagnetic manipulation, the prevailing reliance on time‐harmonic analysis has led most studies to overlook time as a critical control parameter. When the time varying property is treated as an additional design parameter in electromagnetic engineering, the resulting framework offers much greater flexibility and allows effective control of electromagnetic signal characteristics across the space, time, and frequency domains [1, 2]. The curiosity and interest in time‐varying electromagnetic platforms can be traced back several decades. In fact, linear time‐varying circuits have captured the attention of researchers since the 1950s. In microwave device design, parametric amplifiers [3] and time‐modulated nonreciprocal components [4, 5, 6] are classic examples of applications that exploit time‐varying properties.

In terms of aspects more related to electromagnetic or light interaction and matter, phase modulators based on the Pockels effect [7], light amplification in photonic time crystals [8], and the inverse prism based on temporal discontinuity and spatial dispersion [9] have been around for quite some time. In the domain of electromagnetic manipulation, space–time modulation architectures introduce this time varying property into conventional antenna array architecture, providing markedly enhanced control flexibility and enabling effective control of signal distributions across the space, time, and frequency domains [10, 11]. One prominent example is the integration of space–time modulation with metasurface platforms, which has demonstrated significant performance advantages in electromagnetic manipulation [12, 13, 14, 15] and wireless communications [16, 17, 18, 19, 20].

As wireless networks become increasingly intertwined with critical infrastructures, the surrounding electromagnetic environment grows more congested and contested, placing stringent demands on the ability of wireless systems to precisely manipulate radiated fields [21]. In typical operating scenarios, signals emitted by wireless transmitters remain inherently exposed and can be received by unintended observers, potentially leading to interference, information leakage, or even system disruption [22, 23, 24]. This vulnerability has motivated the development of physical‐layer security strategies, which seek to suppress information leakage directly at the level of electromagnetic radiation rather than relying solely on higher‐layer encryption [25, 26, 27, 28, 29]. A practical manifestation of this concept is the reduction of waveform observability at unintended receivers, whereby radiation and waveform characteristics are deliberately engineered to limit observability while preserving the intended system functionality [30].

Achieving physical‐layer security in this sense requires precise regulation of electromagnetic signal characteristics across spatial directions [31]. Owing to its inherently flexible, multi‐dimensional control over electromagnetic radiation, space–time modulation offers a compelling route toward this objective [32, 33, 34, 35]. Recent studies have exploited time‐modulation harmonics for selective multibeam formation and sideband‐controlled beam scanning, and have further extended time‐modulated beam scanning to directional jamming mitigation [36, 37, 38]. Despite these advances, the radiation behavior of existing approaches remains primarily governed by hardware‐level temporal switching. In practical implementations, however, expanding the available radiation‐control degrees of freedom to accommodate complex operating scenarios typically demands a substantial increase in time‐modulation hardware complexity, for example, through the introduction of additional switching elements to enable finer phase control [39, 40, 41, 42, 43]. In large‐scale antenna arrays, such growth in hardware complexity rapidly becomes prohibitive, limiting scalability and practical deployment. Meanwhile, the abundant programmable degrees of freedom available in digital baseband waveform synthesis remain largely unexploited in controlling spatial radiation. Establishing a mechanism for mapping these baseband degrees of freedom into the electromagnetic radiation domain is therefore essential for expanding radiation‐control capability without a corresponding increase in RF hardware complexity.

In this article, we introduce a digitally mediated space–time modulation strategy that coherently couples digital baseband waveform synthesis with analog time modulation. Rather than simply cascading an independent baseband‐processing stage with a conventional time‐modulated array, the proposed strategy constructs the composite baseband waveform according to prescribed periodic time sequences. Their synchronized interaction establishes a deterministic mapping from signal‐domain waveform components to element‐level excitations and ultimately to spatial radiation behavior. This mapping transfers programmable baseband degrees of freedom into the radiation domain, thereby expanding the effective radiation‐control capability beyond that directly provided by hardware‐level switching states, without increasing the resolution or complexity of the modulation network. As a representative demonstration, multiple beams are configured to independently convey different waveform signals toward distinct spatial directions. To validate the proposed strategy, a space–time modulation architecture employing one‐bit phase control is implemented and experimentally characterized. The measured results show that prescribed waveform signals are accurately radiated toward the desired directions, whereas noise‐like emissions dominate the remaining angular regions, effectively obscuring identifiable signal features.

## 2 Results

### 2.1 Digitally Mediated Space‐Time Modulation Scheme

**FIGURE 1:**
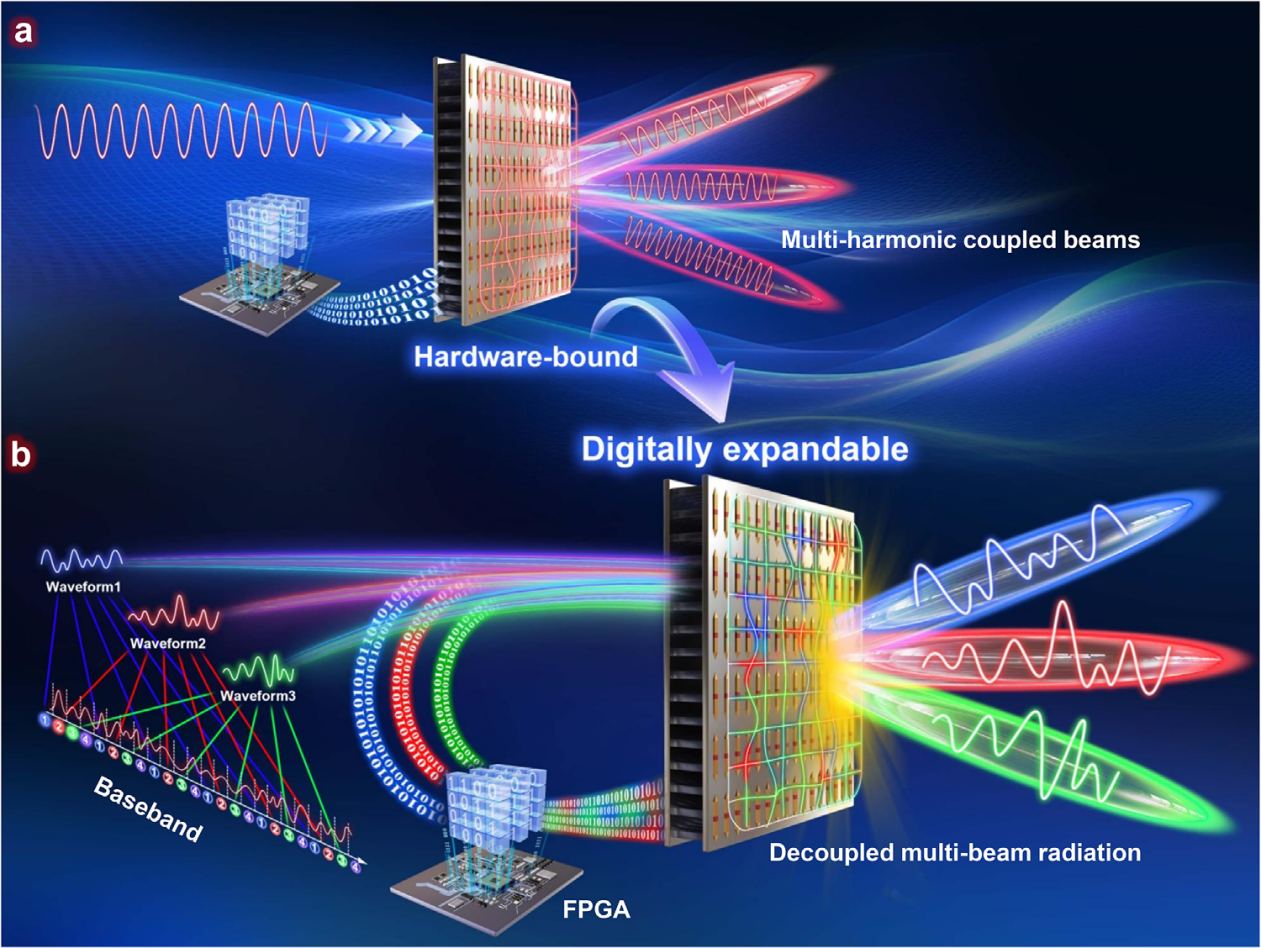
From hardware‐bound space–time modulation to digitally mediated radiation control. (a) Conventional space–time modulation architectures rely on hardware‐level switching to generate multi‐harmonic radiation, in which the available radiation‐control degrees of freedom are inherently constrained by the modulation network and the number of switching states, resulting in limited and coupled harmonic beam patterns. (b) The proposed digitally mediated space–time modulation architecture shifts radiation control from a hardware‐bound paradigm to the digital domain. Multiple waveform signals intended for different spatial directions are digitally mediated at the baseband, where information‐bearing and auxiliary components are jointly orchestrated. Through this digital mediation, the composite signals are coherently coupled to analog time modulation and projected onto predefined spatial regions, enabling flexible and programmable multi‐beam radiation using a simple periodic time sequence.

In conventional space–time modulation architectures (Figure 1a), radiation manipulation is primarily governed by the temporal switching patterns imposed on the antenna elements. As a result, the available degrees of freedom are intrinsically tied to the resolution and complexity of the time‐modulation hardware, such as the number of switching states or phase quantization levels. Increasing radiation‐control capability therefore typically relies on introducing finer time resolution or higher‐bit switching, which directly translates into increased hardware complexity. By contrast, the proposed digitally mediated architecture (Figure 1b) extends radiation control beyond hardware‐bound switching. A unified control interface synchronously governs both the digital baseband modulation module and the analog switching network. In the implemented system, a field‐programmable gate array (FPGA) generates the time sequence and distributes it concurrently to the baseband processor and the switching network, ensuring coherent digital–analog coordination. The time sequence drives temporal switching at the analog front end while simultaneously conditioning the baseband signals, intrinsically coupling signal synthesis to the subsequent space–time radiation process.

Through this coordinated mediation, the effective degrees of freedom are expanded from purely temporal switching states to a composite space–time–signal domain, in which waveform characteristics and radiation directions can be jointly designed. The digital and analog controls are therefore functionally interdependent: the prescribed multiple waveform radiation behavior emerges from the synchronized interaction between the baseband components and the element‐level switching sequences, rather than from either operation independently. Importantly, this expansion does not rely on increasing switching states or modulation branches. Instead, digitally processed waveform components are independently mapped onto distinct spatial directions using the same time‐modulation hardware, thereby enabling concurrent multi‐beam control without a proportional increase in hardware complexity.

A central feature of the proposed scheme is the structured generation of the time‐modulation sequence. Unlike conventional approaches that rely on increasingly complex switching to enhance radiation flexibility, the present scheme employs a deterministic periodic sequence deliberately constructed to satisfy beamforming, nulling and masking constraints while remaining compatible with low‐complexity switching hardware. Specifically, the time sequences assigned to different radiation channels are designed to satisfy a periodic orthogonality condition, such that the product of any two distinct channel sequences integrates to zero over one modulation period (See Section 4). This orthogonality ensures that, after modulation, each channel preferentially extracts its intended signal component while suppressing interference from other beams. In this manner, digitally synthesized waveform constituents can be selectively projected toward prescribed spatial directions. Detailed analytical derivations and optimization procedures for the signal model and orthogonality conditions are provided in Section S1.

It is also important to distinguish the proposed approach from hybrid digital–analog beamforming [44]. In hybrid beamforming architectures, digital and analog processing cooperate primarily at the level of beamforming weight coefficients, with the analog network approximating digitally optimized spatial weights to form a single composite beam. The underlying radiation mechanism therefore remains within the paradigm of conventional static phased arrays. In contrast, the present work employs digital mediation to directly expand the space–time radiation degrees of freedom. By intrinsically coupling digital baseband processing with time‐modulated switching, the analog domain is enabled to generate simultaneous multiple beams with distinct waveform characteristics, representing a fundamentally different radiation mechanism beyond conventional beam steering.

To place these distinctions in a broader context, Table 1 compares the proposed architecture with representative state‐of‐the‐art space–time modulation approaches in terms of functional capability, implementation requirements, and security‐related performance. Although existing architectures provide selected capabilities, such as programmable waveform–beam mapping, distinct waveform assignment or reduced waveform observability, none simultaneously combines decoupled multi‐beam synthesis, dedicated waveform assignment, programmable waveform–beam mapping and a dedicated masking beam. In contrast, the proposed architecture integrates these capabilities while retaining simple periodic time sequences, 1‐bit phase modulation and low hardware overhead. The comparison therefore highlights that the principal contribution of this work lies not in any single capability considered independently, but in unifying programmable multi‐beam waveform synthesis and physical‐layer security within a low‐complexity digitally mediated architecture. The resulting multi‐directional waveform‐mapping and physical‐layer security capabilities are further evaluated in the following sections through numerical simulations and experimental measurements.

**TABLE 1:** Comparison with the state‐of‐the‐art space–time modulation architectures.

	Capability			Implementation			Security	
References	Decoupled multi‐beam synthesis	Distinct waveform per beam	Programmable waveform‐beam mapping	Periodic time sequence	Modulation type	Low hardware overhead	Reduced waveform observability	Dedicated masking beam
*Nat. Electron*. (2022) [14]	×	√	√	√	1‐bit	√	×	×
*Adv. Mater*. Technol. (2019) [18]	×	×	√	×	2‐bit	×	×	×
*Nat. Electron*. (2025) [20]	×	×	√	×	1‐bit	√	×	×
*Nat. Electron*. (2021) [25]	×	×	√	√	1‐bit	√	√	×
*Nat. Commun*. (2025) [27]	×	√	√	√	2‐bit	×	√	×
*Nat. Commun*. (2025) [28]	×	×	√	×	1‐bit	√	√	×
*IEEE TAP* (2025) [34]	√	√	√	×	1‐bit	×	×	×
*IEEE JSTSP* (2016) [44]	√	√	×	N.A.	N.A.	√	×	×
This work	√	√	√	√	1‐bit	√	√	√

### 2.2 Multi‐Directional Waveform Mapping

To elucidate how the expanded radiation‐control degrees of freedom translate into new radiating behaviors, we first examine the capability of the proposed architecture to synthesize direction‐dependent waveform characteristics. Owing to the digitally mediated orchestration between baseband modulation and time modulation, waveform synthesis is no longer globally constrained across space, but can instead be tailored independently for different spatial directions, where such spatial decoupling is not available for conventional space–time modulation architectures. As a result, multiple information‐bearing waveforms can be simultaneously projected toward their intended directions, while their spectral and temporal characteristics become intrinsically distorted elsewhere. Numerical simulations are presented below to demonstrate this direction‐selective waveform synthesis.

**FIGURE 2:**
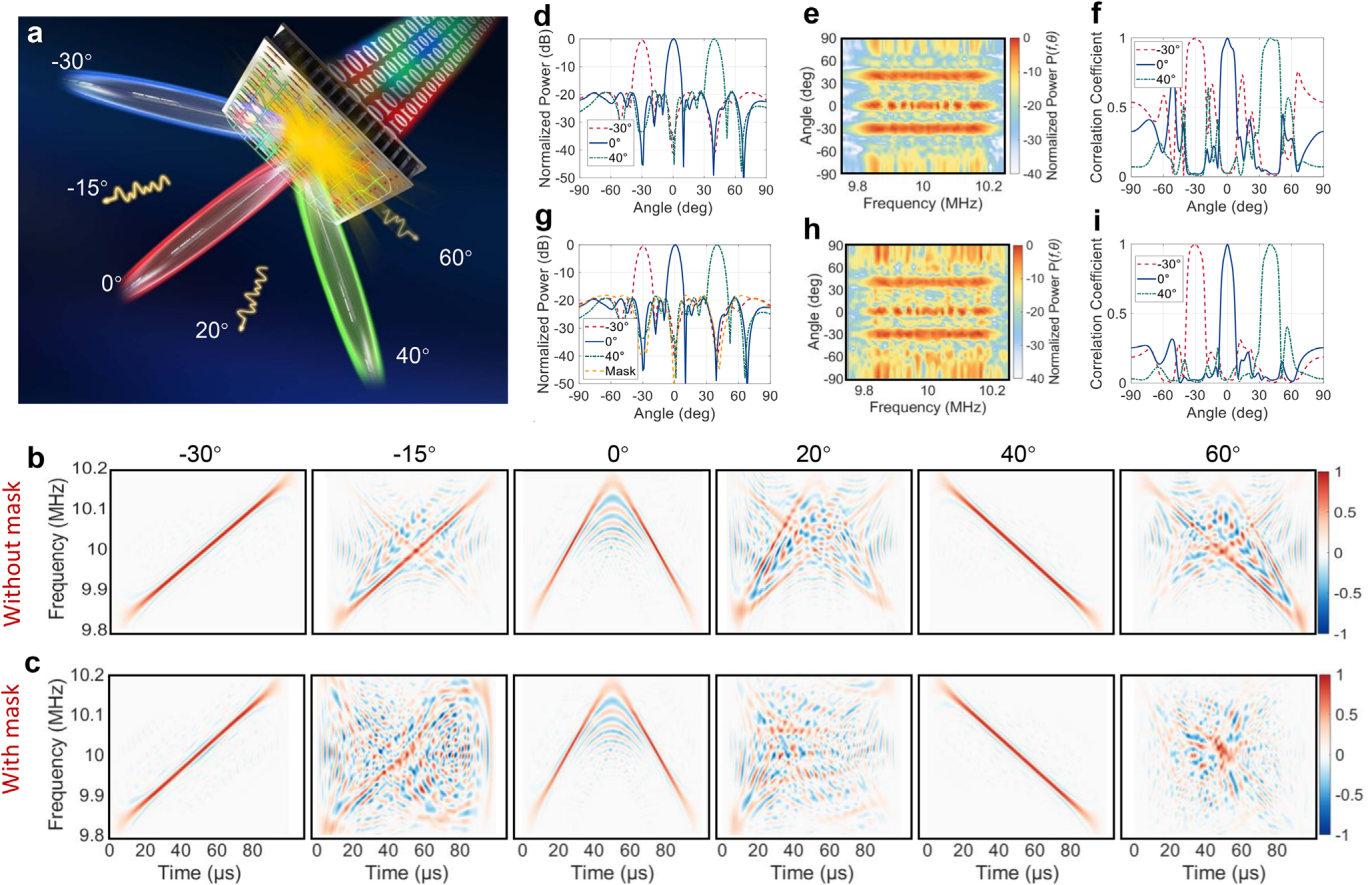
Simulated radiation behavior enabled by digitally mediated space–time modulation. (a) Selected observation angles of −30°, 0°, 40°, −15°, 20° and 60°. (b,c) Time–frequency representations of the radiated signals at the selected angles before (b) and after (c) activating the masking beam, where the vertical axis denotes the intermediate frequency (IF). (d–f) Radiation characteristics before activating the masking beam: simultaneous multi‐beam pattern (d), radiated power distribution in the angle–frequency domain (e), and correlation coefficients of the corresponding time–frequency representations (f). (g–i) Radiation characteristics after activating the masking beam: simultaneous multi‐beam pattern (g), radiated power distribution in the angle–frequency domain (h), and correlation coefficients of the corresponding time–frequency representations (i).

Without loss of generality, the proposed approach generates three directional beams that are independently steered toward −30°, 0°, and 40°, respectively. Then, the three directional beams are individually loaded with different modulation waveforms and radiated through the proposed architecture. Three typical detection waveforms are selected for validation: the positive‐slope linear frequency‐modulated continuous wave (LFMCW), the symmetrical triangular LFMCW, and the negative‐slope LFMCW. The detailed simulation setup is reported in Section S3. The end‐to‐end signal‐processing flowchart is summarized in Supplementary Figure S1. The schematic illustration of the simulation scenario and the corresponding numerical results are shown in Figure 2.

To gain a more intuitive view of the radiated waveforms beyond conventional spectral descriptions, time–frequency analysis is used to visualize how signal features evolve jointly in time and frequency across different spatial directions. Figure 2b illustrates the time−frequency distributions of signals transmitted by the proposed architecture in the directions of −30°, 0°, 40°, −15°, 20°, and 60°. As illustrated, the highlighted shapes in the intended direction of −30°, 0°, and 40° clearly demonstrate the desired signal characteristics of positive‐slope, symmetrical triangular, and negative‐slope LFMCW signals, respectively. In addition, in the unintended directions of ‐15°, 20°, and 60°, although partial signal aliasing is observed, the radiated signals still exhibit discernible characteristics of the three LFMCW waveforms. Figure 2d shows the resulting radiation beam patterns, in which three well‐defined directional beams with low sidelobe levels are formed and accurately steered toward ‐30°, 0°, and 40°, respectively. The sidelobe levels are close to ‐18 dB. Because equal gain targets are assigned to the intended beams during beam‐pattern optimization, their normalized peak gains are approximately equal. The beamwidths and their variations with scanning direction are mainly determined by the effective projected aperture and the active element pattern. It is also noteworthy that in the desired directions of −30°, 0°, and 40°, all beams except those inherently steered toward each respective direction are configured to null. In this way, the proposed approach can minimize mutual interference between the beams, thereby ensuring optimal detection performance. The radiated power distribution is depicted in Figure 2e. It can be observed that three different bright bands emerge in the direction of −30°, 0°, and 40°. Obviously, these bands exhibit clear characteristics of LFMCW signals. However, some residual spectral components exhibit higher local power levels than the sidelobes observed in the corresponding spatial radiation‐pattern cuts. This is primarily attributed to residual coupling and coherent superposition among multiple waveform components, which can locally enhance certain frequency components. Additionally, it can also be observed that the spectrum appears somewhat distorted in the undesired directions, primarily due to signal aliasing caused by overlapping beams carrying different signals. Generally, for signals with multiple frequency components superimposed, time‐frequency distributions can provide detailed information on how each frequency component changes over time, while time‐frequency correlation can reveal the similarity between signals at each frequency component. The calculated correlation coefficients between the time‐frequency distributions of the signals in the desired directions (–30°, 0°, and 40°) and those in the undesired directions are presented in Figure 2f. The higher the correlation coefficient, the greater the similarity between the time‐frequency characteristics of the signals in the two directions. As seen from Figure 2f, multiple high‐correlation peaks emerge across various directions other than the desired one, indicating a significant likelihood of signal interception by an adversary in those directions. It is worth noting that the spectral aliasing observed in the radiated signals constitutes a fundamental mechanism by which conventional space–time modulation architectures enhance transmission security. Such aliasing can be effective in degrading communication quality or hindering demodulation at unintended receivers [25]. However, spectral aliasing alone is insufficient for concealing waveform characteristics. As shown in Figure 2f, even in the presence of aliasing, distinctive time–frequency features of the original waveforms can remain partially identifiable, particularly when advanced signal analysis is employed.

This limitation motivates the introduction of an additional masking beam in the proposed architecture. Rather than relying solely on harmonic aliasing, the masking beam actively introduces controlled radiation irregularity in unintended spatial regions, suppressing waveform identifiability at the feature level. In this manner, the masking beam complements the inherent spectral aliasing of space–time modulation and enables a more stringent form of radiation‐domain physical‐layer security based on waveform concealment rather than demodulation impairment alone.

### 2.3 Physical‐Layer Security Behavior

This section presents a quantitative evaluation of the resulting physical‐layer security behavior, focusing on how the masking beam suppresses waveform observability in unintended spatial regions. Unlike the information‐bearing beams, the masking beam does not serve a sensing or communication function. Instead, it is deliberately shaped through joint digital baseband processing and time modulation to occupy unintended angular regions and introduce controlled radiation irregularity. By superimposing noise‐like or decorrelated waveform components onto these regions, the masking beam obscures identifiable signal features without perturbing the desired beams. The detailed design procedure and optimization strategy of the masking beam are provided in Section S2. In this manner, physical‐layer security emerges as a radiation‐domain behavior that is intrinsically coupled to the digitally mediated space–time modulation process.

With the masking beam activated, the time–frequency representations of the radiated signals observed at −30°, 0°, 40°, −15°, 20°, and 60° are shown in Figure 2c. Along the intended directions of −30°, 0°, and 40°, the waveform characteristics are preserved with minimal distortion. By contrast, the signals observed in the unintended directions of −15°, 20°, and 60° exhibit pronounced spectral and temporal distortion, rendering the waveform features difficult to identify.

Figure 2g illustrates the radiation patterns after activating the masking beam. When the masking beam is radiated concurrently, the three directional beams remain essentially unchanged, indicating that the introduction of the masking radiation does not degrade the integrity of the original directional beams. In contrast to a conventional directional beam, the masking beam exhibits an irregular radiation profile. As shown in Figure 2g, this irregular beam effectively spans nearly all unintended angular regions while leaving the desired directions largely unaffected. By loading noise‐like masking signals onto this beam, the waveform characteristics in unintended directions are strongly obscured, leading to a pronounced suppression of waveform observability and thereby enhancing radiation‐domain physical‐layer security. As reflected in the radiated power distribution in Figure 2h, the concurrent activation of the masking beam further intensifies signal distortion in unintended spatial regions. In parallel, the introduction of the masking radiation markedly suppresses the correlation coefficients of the time–frequency distributions over most unintended directions, while those associated with the desired directions remain largely unaffected, as shown in Figure 2i.

Through this spatially selective distortion enabled by the masking beam, the proposed architecture achieves a substantial suppression of waveform observability in unintended regions, thereby significantly enhancing radiation‐domain physical‐layer security. In this work, waveform observability is used as the primary metric for the LFMCW demonstration, while communication‐level demodulation performance is evaluated using error vector magnitude (EVM) in Section S3.3. This criterion is more stringent than conventional secure communication metrics, as reliable waveform identification is a prerequisite for subsequent operations such as synchronization, parameter estimation and demodulation. If the characteristic features of a signal cannot be easily identified, successful interception and decoding become more challenging. Consequently, the demonstrated suppression of waveform observability provides a conservative indicator of secure transmission, confirming the effectiveness of the proposed digitally mediated space–time modulation framework in achieving radiation‐domain physical‐layer security.

### 2.4 Experimental Demonstration of Digitally Mediated Space–Time Modulation

**FIGURE 3:**
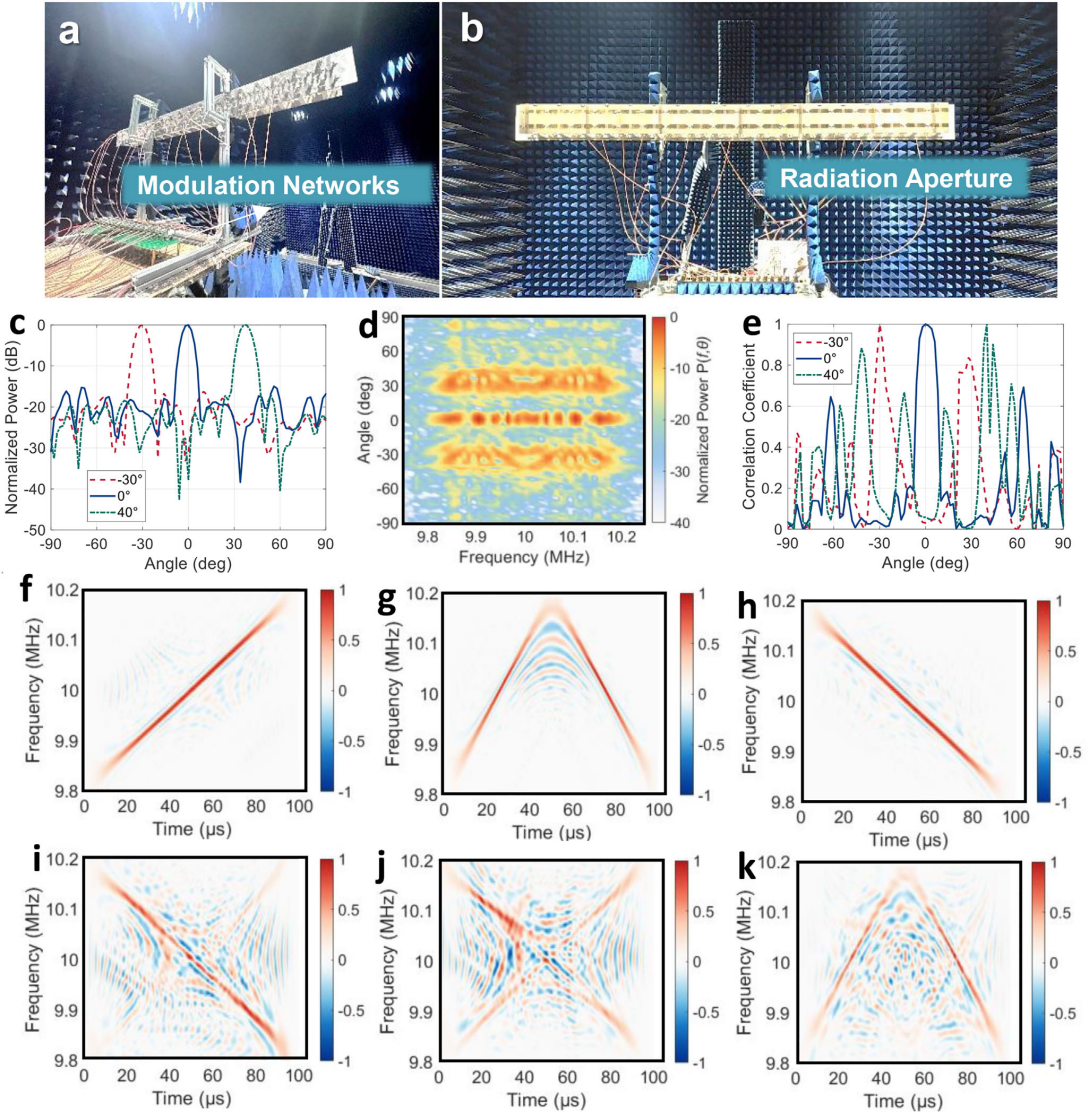
Experimental validation of digitally mediated space–time modulation. (a,b) Photographs of the implemented prototype. (c) Measured simultaneous multi‐beam radiation pattern. (d) Measured radiated power distribution in the angle–frequency domain. (e) Measured correlation coefficients of the corresponding time–frequency representations. (f–k) Time–frequency distributions of the measured signals observed at −30° (f), 0° (g), 40° (h), −15° (i), 20° (j), and 60° (k).

To verify that the expanded radiation‐control degrees of freedom and the associated suppression of waveform observability persist, an experimental prototype of the digitally mediated space–time modulation architecture was constructed and characterized. The prototype employs a simple switching network with one‐bit phase control in a 16‐element array, together with synchronized digital baseband processing, to realize joint digital–analog mediation under practical hardware constraints (See Section 4 for the detailed measurement setup). Using this experimental platform, we examine whether direction‐dependent waveform synthesis and spatially selective waveform obfuscation, as predicted by the simulations, can be physically realized in a radiating system. The measured radiation patterns and waveform characteristics presented below confirm that the proposed architecture achieves the intended space–time modulation behavior while maintaining low hardware complexity. Photographs of the fabricated prototype and the measurement configuration are shown in Figure 3a,b. All measurements were performed in a microwave anechoic chamber.

To establish a baseline for beamforming performance in the absence of physical‐layer security considerations, the measured radiation patterns obtained without the masking beam are shown in Figure 3c. Three directional beams are observed to steer accurately toward −30°, 0° and 40°, respectively, with sidelobe levels of approximately −16 dB. The measured patterns exhibit good agreement with the simulated results in Figure 2d in terms of both pointing directions and overall beam shapes. Minor discrepancies can be observed, including differences in relative beam gains, beamwidths, slight angular offsets, and degraded null depths at specific angles. These deviations stem from non‐idealities arising in practical implementation. Nevertheless, the majority of null locations remain consistent with the simulations, confirming the validity of the proposed beamforming mechanism. For example, at 0°, all beams other than the one intentionally directed toward this angle are effectively suppressed. In the proposed architecture, beam formation is jointly governed by the applied time sequence and the digitally mediated baseband signals. Accordingly, the observed deviations primarily arise from non‐ideal switching behavior and nonlinear effects along the signal transmission chain. Despite these imperfections, the experimental results clearly demonstrate the flexible and accurate beamforming capability enabled by the digitally mediated space–time modulation scheme. The measured radiated power distributions in the angle–frequency domain obtained without physical‐layer security measures and hence without the masking beam are shown in Figure 3d. In agreement with the simulation results, three well‐defined regions are observed, each corresponding to a distinct LFMCW waveform projected toward its intended spatial direction.

To quantitatively evaluate the observability of the radiated waveforms, the correlation function of the time–frequency distributions is employed, and the resulting correlation coefficients are presented in Figure 3e. The experimental results closely follow the simulated trends. Compared with the simulations, the measured correlation coefficients exhibit slightly sharper peaks, which are attributed to spurious spectral components and practical non‐idealities in the experimental system. Notably, when only the three directional beams are radiated, waveform characteristics over most angular regions remain highly correlated with the original signals, indicating that the radiated waveforms remain readily identifiable at unintended spatial locations in the absence of masking.

To further characterize the baseline waveform observability in the absence of physical‐layer security measures, representative spatial directions of −30°, 0°, 40°, −15°, 20° and 60° are also selected for detailed analysis of the time–frequency signal distributions. The corresponding results obtained without the masking beam are shown in Figure 3f–k. Along the intended directions of −30°, 0°, and 40°, the characteristic features of the three LFMCW waveforms are well preserved, confirming faithful waveform delivery toward the targets. By contrast, in the unintended directions of −15°, 20°, and 60°, the LFMCW signatures remain clearly discernible despite the presence of moderate interference. This persistence of identifiable waveform features indicates that, under this baseline condition, the radiated signals in unintended regions remain readily observable, offering limited radiation‐domain physical‐layer security. These observations establish a reference case against which the impact of the digitally mediated masking beam on waveform obfuscation and the suppression of waveform observability is examined below.

**FIGURE 4:**
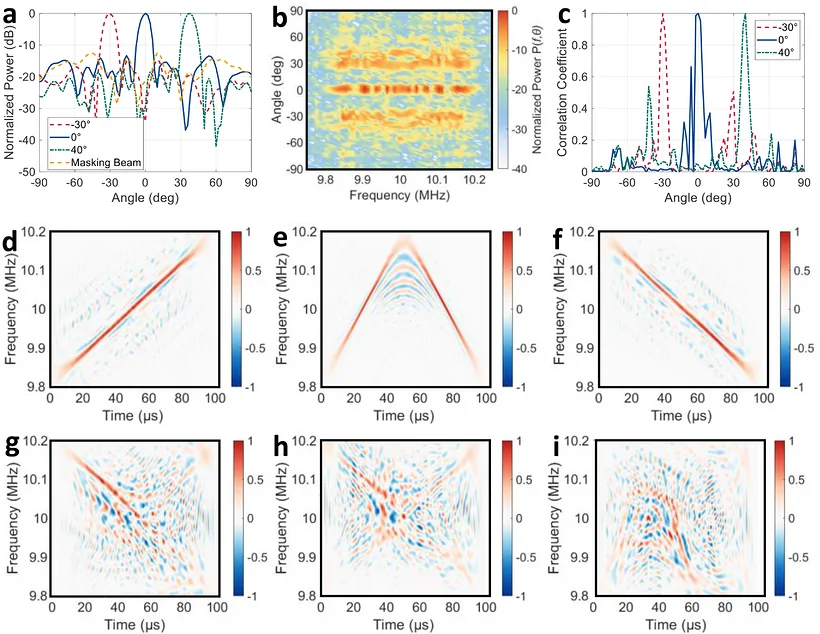
Experimental radiation behavior with digitally mediated masking enabled. (a) Measured simultaneous multi‐beam radiation pattern. (b) Measured radiated power distribution in the angle–frequency domain. (c) Measured correlation coefficients of the corresponding time–frequency representations. (d–i) Time–frequency distributions of the measured signals observed at −30° (d), 0° (e), 40° (f), −15° (g), 20° (h), and 60° (i).

When the digitally mediated masking beam is activated in the proposed architecture, the corresponding experimental results are shown in Figure 4. The measured radiation patterns are presented in Figure 4a. Despite the presence of the masking beam, the three directional beams retain nearly identical shapes and pointing directions relative to the baseline case, indicating that each beam remains independently controllable and that inter‐beam coupling is negligible. At the same time, the masking beam spans the unintended angular regions, enabling effective distortion of the radiated signal characteristics outside the target directions. The measured angle–frequency power distribution with the masking beam enabled is shown in Figure 4b. In contrast to the baseline case, the radiated power exhibits a more irregular and disordered distribution across unintended spatial regions, reflecting the superposition of information‐bearing and masking components and creating favorable conditions for the suppression of waveform observability. The corresponding correlation coefficients of the time–frequency distributions are plotted in Figure 4c. When the directional beams and the masking beam are radiated concurrently, the correlation between the received waveforms in unintended directions and the original signals is markedly reduced, demonstrating a substantial enhancement in radiation‐domain physical‐layer security.

Following the same analysis procedure, representative spatial directions of −30°, 0°, 40°, −15°, 20°, and 60° are examined with the digitally mediated masking beam activated, and the corresponding time–frequency distributions are shown in Figure 4d–i. Along the intended directions of −30°, 0°, and 40°, the characteristic features of the three LFMCW waveforms remain well preserved, confirming reliable waveform delivery toward the targets. By contrast, in the unintended directions of −15°, 20°, and 60°, the radiated signals are effectively obscured by the masking component, rendering their waveform features difficult to identify. This pronounced reduction in waveform discernibility in unintended spatial regions directly demonstrates the suppression of waveform observability and thereby confirms the enhanced radiation‐domain physical‐layer security enabled by the proposed digitally mediated space–time modulation architecture.

These observations indicate that digitally mediated masking provides a flexible mechanism for spatial waveform concealment under practical uncertainty in unintended receiver locations. Further supporting results are provided in Section S3. Specifically, Section S3.1 describes the simulation configuration adopted for the numerical analyses; Section S3.2 presents additional LFMCW simulations for an alternative set of prescribed beam directions at −45°, 10°, and 35°, including the optimized time sequence and digital weights, multi‐beam patterns, radiated‐power distributions, time–frequency responses, and correlation analysis (Figures S2–S8), thereby confirming the robustness of the proposed method across representative angular configurations; and Section S3.3 extends the validation to OFDM–QAM (orthogonal frequency division multiplexing–quadrature amplitude modulation) communication waveforms, with simulated angle–frequency power distributions, angular EVM values, and constellation diagrams shown in Figures S9 and S10 and the corresponding experimental results shown in Figures S11 and S12. Together, these results further demonstrate the waveform versatility and direction‐dependent protection capability of the proposed framework.

## 3 Conclusions

This study establishes digitally mediated space–time modulation as a new paradigm for controlling electromagnetic radiation in complex environments. Its central advance lies in establishing a mediated coupling between digital baseband waveform design and time‐modulated radiation, rather than merely adding an independent baseband‐processing stage to a conventional 1‐bit time‐modulated array. By enabling programmable baseband degrees of freedom to participate directly in electromagnetic‐field synthesis and coherently coordinating them with time modulation, the proposed framework extends radiation‐control capability beyond that directly provided by hardware‐level switching states. In particular, it allows simultaneous multi‐beam waveform synthesis together with intrinsic suppression of waveform observability in unintended spatial regions, without incurring the hardware complexity traditionally associated with multi‐bit modulation. Beyond the specific experimental demonstration presented here, this concept suggests a scalable pathway toward programmable, secure, and multifunctional radiating systems. Broader considerations regarding scalability, hardware constraints and potential limitations are discussed in Supplementary Section S5. As sensing and communication infrastructures continue to converge and operate under increasingly contested spectral conditions, digitally mediated control of space–time radiation may provide a foundational mechanism for future adaptive electromagnetic platforms.

## 4 Methods

The following section describes the implementation of the proposed digitally mediated space–time modulation framework and the procedures used for numerical evaluation and experimental validation. Emphasis is placed on the architectural realization of joint digital–analog mediation, the generation and synchronization of digital baseband and time‐modulation signals, and the configuration of the experimental prototype under practical hardware constraints.

### 4.1 Digitally Mediated Space–Time Modulation Framework

**FIGURE 5:**
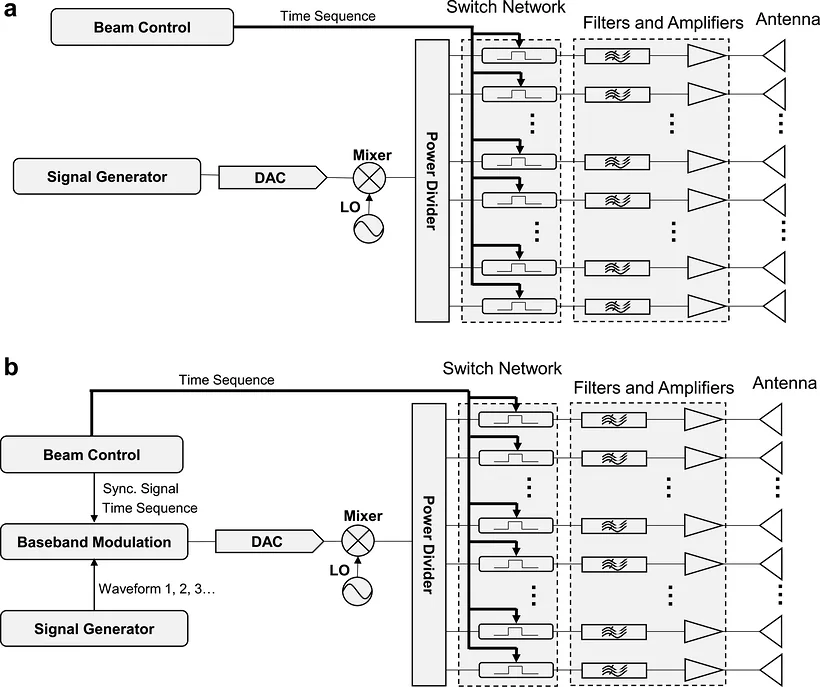
Hardware architecture comparison of space–time modulation systems. (a) Conventional space–time modulation architecture. The RF signal is distributed to the time‐modulated switching network, where beamforming is governed solely by the switching sequence before radiation by the antenna array. (b) Digitally mediated space–time modulation architecture. Waveform information generated by the signal generator is processed in the digital baseband modulation module and converted to an analogue baseband signal by a digital‐to‐analogue converter (DAC). The signal is then up‐converted to radio frequency by a mixer and distributed by a power divider to the time‐modulated switching network. After time modulation, the signals are filtered to suppress out‐of‐band components, amplified, and radiated by the antenna array. For digitally mediated control, the beam control module interfaces simultaneously with the baseband modulation module and the switching network. A time sequence is generated according to the prescribed beamforming configuration and delivered concurrently to both subsystems, enabling synchronized baseband modulation and time‐modulated switching.

As illustrated in Figure 5, Figure 5a depicts a conventional space–time modulation architecture, in which radiation behavior is governed exclusively by the temporal switching sequence of the time‐modulated network. In this configuration, the baseband signal is treated as a passive input, and spatial radiation characteristics are determined solely by the switching operation. By contrast, Figure 5b presents the proposed digitally mediated architecture, in which radiation control is achieved through the coordinated interaction of digital baseband processing and time‐modulated switching. A beam control unit interfaces simultaneously with the baseband processor and the switching network, enabling synchronized digital–analog mediation. Under this unified control, a time sequence is generated according to the prescribed beamforming configuration and applied not only to the switching network for time modulation, but also to the baseband module to govern waveform synthesis. In this manner, baseband signal formation and space–time radiation are intrinsically coupled. Following time modulation, the signals are filtered, amplified and radiated by the antenna array.

In this framework, the baseband signals define the amplitude, phase, and waveform composition associated with each beam, while the time‐modulation sequence governs the temporal switching of the array elements. In this manner, the radiated field is governed by a set of digitally controllable variables, including the baseband waveform composition and digital weighting coefficients, together with a time‐modulation sequence constrained by the switching hardware. The time sequence simultaneously mediates digital baseband processing and analog switching, establishing a direct mapping between digitally defined signals and their space–time radiation behavior. Based on this formulation, the following analysis develops a sequence of key expressions that progressively describe how the digitally defined baseband signals and the time‐modulation sequence jointly determine the resulting space–time radiation behavior.

The baseband signal is defined as the sum of multiple original waveform signals following digital pre‐modulation, and it can be mathematically expressed as

(1) $$s\left(t\right)=\sum_{p=1}^{Q}s_{p}\left(t\right)\cdot h_{p}\left(t\right)$$

where *s_p_* (*t*) denotes the *p‐*th original baseband signal. *Q* represents the number of original baseband signals, and *h_p_* (*t*) is the baseband pre‐modulated signal corresponding to the *p‐*th signal *s_p_* (*t*). When introducing design degrees of freedom in baseband, the key focus primarily lies in the design of the pre‐modulation signal *h_p_* (*t*). For flexible and secure beamforming, the pre‐modulated signal *h_p_* (*t*) is defined as an explicit function of the applied time sequence *U_k_* (*t*). This relationship can be expressed as

(2) $$h_{p}\left(t\right)=\sum_{k=1}^{N}U_{k}\left(t\right)\cdot A_{p}^{k}e^{j\varphi_{p}^{k}}$$

where $A_{p}^{k}$ and $\varphi_{p}^{k}$ represent the digital amplitude and phase weighting coefficients, respectively. From the above expression, it can be observed that, unlike in conventional space–time modulation arrays where *s*(*t*) represents only the original baseband waveform to be transmitted, in the proposed architecture *s*(*t*) corresponds to a pre‐modulated composite signal. Within this framework, suppression of waveform observability is achieved by jointly generating information‐bearing beams for sensing and dedicated masking beams for spatial waveform concealment. To realize this functionality, the applied time‐modulation sequence should satisfy the following condition:

(3) $$\int_{-\frac{T_{s}}{2}}^{\frac{T_{s}}{2}}U_{k}\left(t\right)\cdot U_{l}\left(t\right)dt=\left\{\begin{array}{c} T_{s},\;\;k=l\\ 0,\;\;k\neq l \end{array}\right.$$

where *U_k_* (*t*) denotes the time sequence function for the *k‐*th element at time *t*. Therefore, when the radiated signal from the proposed architecture at the central frequency passes through the band‐pass filter, it can be mathematically expressed as

(4) $$E_{t}\left(\theta ,t\right)\;=e^{j2\pi f_{0}t}\sum_{p=1}^{Q}s_{p}\left(t\right)\cdot \sum_{k=1}^{N}A_{p}^{k}e^{j\varphi_{p}^{k}}\cdot e^{-j\beta (k-1)d\sin\theta }$$

By observing the above formula, it can be found that each original baseband signal is transmitted through a beam characterized by its unique amplitude and phase weightings. In particular, $A_{p}^{k}$ and $\varphi_{p}^{k}$ denote the amplitude weighting factor and phase weighting factor, respectively, of the *p*‐th beam at the *k‐*th element. Like conventional space‐time modulated architectures [45, 46, 47], the proposed architecture generates non‐zero‐order harmonics and therefore involves an intrinsic spectral‐power trade‐off. The complete quantitative power analysis is provided in Section S1.2. From an architectural perspective, the proposed framework separates signal‐domain synthesis from hardware‐level switching and reconnects them through synchronized control. This separation allows radiation behavior to scale primarily with digital processing capability rather than with switching‐state complexity. As a result, the effective radiation‐control dimensionality can be expanded without proportionally increasing hardware resolution, providing a structurally different pathway from conventional hybrid beamforming architectures.

Building on this digitally mediated control, the architecture supports multi‐directional radiation with reduced waveform observability in unintended spatial regions. Multiple information‐bearing beams are generated to deliver prescribed waveforms toward their intended directions, while a dedicated masking beam is concurrently formed to span the remaining angular regions and introduce controlled radiation irregularity. To preserve waveform integrity and minimize inter‐beam coupling, nulling constraints are uniformly imposed across all beams along the intended directions. This design strategy enables concurrent control of beam directionality and waveform concealment without increasing time‐modulation hardware complexity. The resulting reduction in waveform observability should not be interpreted as absolute undetectability or unconditional secrecy. Rather, it is intended to increase the observation time, receiver resources, prior knowledge and computational effort required for signal acquisition, separation and decoding in unintended directions, thereby making timely recovery of useful information more difficult. Advanced multi‐antenna reception, blind source separation or machine‐learning‐based processing may still achieve eventual recovery under sufficiently favorable conditions. Accordingly, absolute immunity against such interception cannot be guaranteed. The above expressions capture the essential signal relationships underlying the proposed digitally mediated space–time modulation framework. Detailed signal‐model derivations, including the eavesdropping model, worst‐case signal‐to‐interference‐plus‐noise ratio (SINR), achievable secrecy‐rate analysis, and their applicability boundaries, are provided in Section S1, while the corresponding parameter‐optimization strategies are described in Section S2.

### 4.2 Numerical Simulation Setup

Numerical simulations were performed to evaluate the radiation behavior enabled by the proposed digitally mediated space–time modulation framework. Three simultaneous directional beams were synthesized and steered toward −30°, 0°, and 40°, respectively. In addition, a dedicated masking beam was generated to span the unintended angular region for the purpose of suppressing waveform observability. Each of the three directional beams was assigned a distinct waveform and radiated through the proposed architecture. Specifically, three commonly used detection waveforms were considered: a positive‐slope LFMCW, a symmetric triangular LFMCW, and a negative‐slope LFMCW. The masking beam was loaded with noise‐like signals to maximize waveform concealment in unintended spatial regions. All numerical results in Figure 2 were obtained using a 16‐element linear array with half‐wavelength element spacing at 1.9 GHz and 0°/180° phase modulation.

**FIGURE 6:**
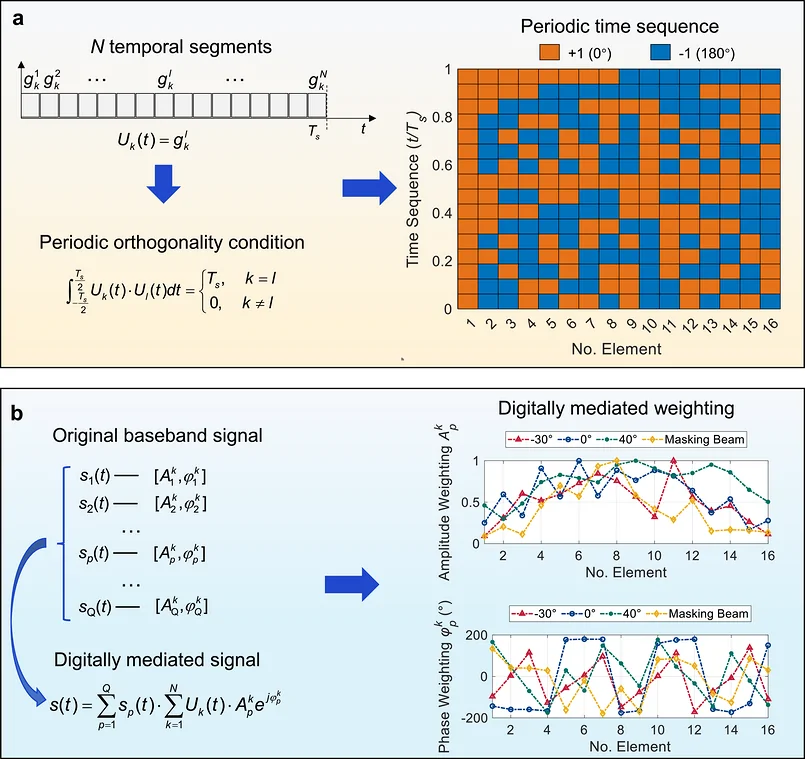
Time modulation and digital weighting underlying digitally mediated space–time modulation. Structured time sequences (a) are combined with digitally optimized amplitude and phase coefficients (b) to enable independent multi‐beam synthesis and masking‐beam formation.

To clarify the underlying time modulation and digital modulation mechanism adopted in these simulations, Figure 6 presents the generated time sequences together with the corresponding digital weighting coefficients. Figure 6a shows the deterministic periodic switching sequences across the array elements within one modulation period, highlighting their structured segmentation and orthogonality. These sequences satisfy the periodic orthogonality condition required for inter‐beam decoupling and are deliberately constructed in a deterministic manner, rather than relying on the pseudorandom switching strategies commonly adopted in conventional low‐observability designs. Figure 6b displays the digitally optimized amplitude and phase weightings associated with the different beams, which define the composite baseband signal prior to time modulation. The structured time modulation and digitally assigned weighting coefficients determine how waveform components are projected into distinct spatial radiation modes.

The joint application of structured time modulation and digital weighting enables distinct waveform components to be projected toward prescribed spatial directions while suppressing undesired interference in unintended regions. Under this controlled configuration, the simulations provide a systematic baseline for evaluating multi‐beam radiation behavior and waveform observability characteristics. Further simulation details and extended numerical results are reported in Section S3.

It is worth noting that conventional space–time modulation architectures are not included as a direct numerical baseline in the present simulations. Under 1‐bit phase modulation, each array element is restricted to two conjugate switching states, which imposes an inherent symmetry on the generated harmonic components [20]. As a result, the radiation‐control degrees of freedom available to conventional architectures are strongly constrained, leading to coupled multi‐beam radiation that is typically symmetric with respect to the broadside direction rather than independently steerable. Consequently, conventional space–time modulation does not readily support the class of independently directed multi‐beam configurations considered here and is therefore not considered as a direct numerical reference under the same hardware constraints.

### 4.3 Experimental Prototype and Measurement Configuration

To experimentally validate the proposed digitally mediated space–time modulation framework, a 16‐element antenna array employing 1‐bit phase control was implemented and characterized. The array is based on a tightly coupled dipole design [48] and provides continuous operation over a bandwidth from 0.5 to 2 GHz. All measurements were conducted at a carrier frequency of 1.9 GHz and an intermediate frequency of 10 MHz. The phase switching network enables time‐modulated transitions between 0° and 180° phase states. The measured transmission phases of the two switching states and their corresponding phase difference are provided in Figure S13. The enhanced radiation‐control flexibility is achieved by shifting part of the implementation burden from the RF hardware to the digital domain, thereby increasing the requirements for baseband computation, memory and data throughput while retaining a simple 1‐bit time‐modulated RF architecture. The coherent digital–analog co‐design also requires deterministic synchronization between the digitally premodulated waveforms and the switching sequences, which is maintained in the present prototype using a common‐clock FPGA. Using this experimental platform, the proposed architecture was configured to generate three simultaneous directional beams, each conveying a distinct waveform. For comparison, measurements were also performed without activating the masking beam, thereby establishing a baseline case for assessing the subsequent suppression of waveform observability enabled by the proposed framework. Detailed experimental procedures and calibration information are provided in Section S4, while the implementation trade‐offs and related scalability considerations are discussed in Section S5. The complete synchronized experimental configuration and signal‐routing chain are shown in Figure S14.

## Author Contributions

S.Y. and K.C. conceived the concept. K.C. and L.Z. developed the theoretical framework and performed the analytical modelling. F.Y. and K.C. designed the digitally mediated space–time modulation architecture and conducted the experimental measurements. G.W. and H.M. contributed to the experimental design and implementation. Y.C., S.W.Q., and J.H. participated in the data analysis and interpretation of the results. S.Y. and J.H. supervised the overall research. K.C., G.W., and L.Z. wrote the manuscript with input from all authors.

## Conflicts of Interest

The authors declare no conflicts of interest.

## Supporting information




**Supporting File**: advs77688‐sup‐0001‐SuppMat.docx.

## Data Availability

The data that support the findings of this study are available from the corresponding authors upon reasonable request.
