# Supplementary material for: Multi‐Dimensional Physical‐Layer Security via Digitally Mediated Space–Time Modulation
Source: Adv Sci (Weinh). 2026 Sep 27:e77688. Online ahead of print. doi: 10.1002/advs.77688 (PMC13616417; doi:10.1002/advs.77688)
Supplement: Supplementary file 1 [file ADVS-9999-e77688-s001.docx]

**Supplementary information for**

**Multi-dimensional physical-layer security via digitally mediated space–time modulation**

Kejin Chen^1^, Heng Mao^1^, Feng Yang^1^, Gengbo Wu^2,*^, Lei Zhang^3,*^, Yikai Chen^1^, Shi-Wei Qu^1^, Jun Hu^1^, and Shiwen Yang^1,*^

^1^ *School of Electronic Science and Engineering, University of Electronic Science and Technology of China (UESTC), Chengdu, China*

^2^ *State Key Laboratory of Terahertz and Millimeter Waves, Department of Electrical Engineering, City University of Hong Kong, Hong Kong, China*

^3^ *State Key Laboratory of Millimeter Waves, School of Information Science and Engineering, Southeast University, Nanjing 210096, China*

### * Corresponding authors: E-mail: bogwu2@cityu.edu.hk, lzseu@seu.edu.cn, swnyang@uestc.edu.cn

### **SI Section S1 — Theoretical formulation and performance analysis**

**S1.1 Basic signal model and derivation**

Fig. **5a** in the main text illustrates a conventional space–time modulation configuration, in which the radiated field can be expressed as the superposition of time-switched element responses driven by an externally supplied baseband signal. In this case, the switching function and the transmitted waveform are separable in the signal model. In contrast, the digitally mediated configuration shown in Fig. **5b** requires a coupled formulation, as the baseband waveform is pre-processed under the same time sequence that governs the switching network. Accordingly, the following derivation establishes a unified analytical model that incorporates both the baseband signal synthesis and the time-modulation operation. This formulation establishes the analytical foundation for the beamforming and masking strategies described in the main text.

For an *N*-element space–time modulated array, the radiated field can be expressed as

$$E(\theta,t)=e^{j2\pi f_{0}t}\sum_{k=1}^{N} U_{k}(t)\cdot e^{-j\beta(k-1)d\sin\theta}$$

where *U_k_*(*t*) denotes the time sequence function for the *k-*th element at time *t*, *f_0_* is the carrier frequency, *β* represents the free space wave number, *d* refers to the spacing between adjacent elements, and *θ* denotes the angle. When *U_k_*(*t*) is treated as a periodic function and the modulation switch is modelled as phase switching (0/180°), the time-domain representation of *U_k_*(*t*) can be formulated as

$$U_{k}(t)=g_{k}^{l},\text{ }g_{k}^{l}=1\text{ }or\text{ -}1,\text{ }T_{s}\frac{l-1}{N}\leq t\leq T_{s}\frac{l}{N}$$

where *T_s_* represents the time modulation period and *l* denotes the discrete time index corresponding to the *l*-th time instant. In this way, through the expansion of the Fourier series, the radiation field of the proposed architecture can be expressed as

$$E(\theta,t)=\sum_{m=-\infty}^{+\infty} e^{j2\pi(f_{0}+mf_{s})t}\sum_{k=1}^{N} a_{mk}e^{-j\beta(k-1)d\sin\theta}$$

where *a_mk_* are the Fourier coefficients. It can be considered as an equivalent complex excitation. In addition, *f_s_* = 1/*T_s_* represents the time modulation frequency, which should be much lower than the carrier frequency (*f_s_* << *f_0_*). The baseband signal is defined as the sum of multiple original waveform signals following pre-modulation, and it can be mathematically expressed as

$$s(t)=\sum_{p=1}^{Q} s_{p}(t)\cdot h_{p}(t)$$

where *s_p_*(*t*) denotes the *p-*th original baseband signal. *Q* represents the number of original baseband signals, and *h_p_*(*t*) is the baseband pre-modulated signal corresponding to the *p-*th signal *s_p_*(*t*). When introducing design degrees of freedom in baseband, the key focus primarily lies in the design of the pre-modulation signal *h_p_*(*t*). For flexible and secure beamforming, the pre-modulated signal *h_p_*(*t*) is defined as an explicit function of the applied time sequence *U_k_*(*t*). This relationship can be expressed as

where $A_{p}^{k}$ and represent the digital amplitude and phase weighting coefficients, respectively. As a result, by substituting (S4) and (S5) to (S1), the radiation field of proposed architecture with baseband signal can be written as

As seen from (S6), the original signal *s_p_*(*t*) has been subjected to dual modulation, encompassing both digital baseband modulation and analog time modulation. To achieve radiation-domain physical-layer security, waveform observability in unintended spatial regions must be minimized. In conventional array architectures, this objective is commonly pursued by employing pseudorandom time sequences to increase signal uncertainty. Digital baseband modulation can further contribute to this process by shaping the transmitted waveforms prior to radiation.

In the proposed framework, suppression of waveform observability is realized by simultaneously generating information-bearing beams for information sensing and dedicated masking beams for spatial waveform concealment. The central design problem therefore becomes how to synthesize multiple directive beams for the intended functions while concurrently producing masking radiation that obscures signal characteristics in non-target directions. For the generation of multiple beams, the applied time sequence must satisfy the following condition:

$$\int_{-\frac{T_{s}}{2}}^{\frac{T_{s}}{2}} U_{k}(t)\cdot U_{l}(t)dt=\left\{\begin{matrix} T_{s},\text{ }k=l \\ 0,\text{ }k\neq l \end{matrix}\right.$$

The radiation field of proposed architecture can also be formulated through the Fourier series expansion as

where $\alpha_{kl}^{m}$ denote the Fourier coefficients at the *m*-th order sideband. Specifically, $\alpha_{kl}^{m}$ can be formulated as

$$\alpha_{kl}^{m}=\frac{1}{T_{s}}\int_{-\frac{T_{s}}{2}}^{\frac{T_{s}}{2}} U_{k}\left(t\right)U_{l}\left(t\right)e^{-j2\pi f_{s}mt}dt$$

When the time sequence satisfies the condition formulated in (S7), it can be found that

$$\alpha_{kl}^{0}=\left\{\begin{matrix} 1,\text{ }k=l \\ 0,\text{ }k\neq l \end{matrix}\right.$$

Therefore, when the radiated signal from the proposed architecture at the central frequency passes through the band-pass filter, it can be mathematically expressed as

By observing the above formula, it can be found that each original baseband signal is transmitted through a beam characterized by its unique amplitude and phase weightings. In particular, $A_{p}^{k}$ and denote the amplitude weighting factor and phase weighting factor, respectively, of the *p*-th beam at the *k-*th element. For convenience, the *p*-th beam can be formulated as

In this manner, the proposed digitally mediated space–time modulation framework establishes a unified mechanism for controlling electromagnetic radiation through the coherent interaction of digital baseband processing and time modulation. By shifting a substantial portion of radiation-control degrees of freedom into the digital domain while maintaining simple time-modulation hardware, the architecture enables programmable beamforming, simultaneous multi-beam waveform synthesis and spatially selective waveform concealment within a single system.

**S1.2 Harmonic-power redistribution and central-frequency modulation efficiency**

The sequence-orthogonality condition established in Supplementary Section S1.1 ensures that the desired composite waveform is recovered in the zero-order component. Nevertheless, the time-domain products of the digital premodulation and switching sequences generally contain non-zero-order Fourier coefficients, thereby redistributing part of the modulation-spectrum power from the central-frequency component to harmonic components centred at $f_{0}+mf_{s}$. To quantify this intrinsic trade-off, we define the central-frequency modulation efficiency as the power contained in the zero-order component divided by the total power distributed over all modulation harmonics.

According to the signal model derived in the previous subsection, the baseband signal can be expressed as

Here, $s_{p}(t)$ denotes the *p*-th signal, $U_{l}(t)$ is the corresponding time sequence, and is the desired complex excitation coefficient of the *p*-th signal at the *l*-th antenna element. Defining

The digital signal can be written more compactly as

$$s(t)=\sum_{l=1}^{N} U_{l}(t)v_{l}(t).$$

After applying the switching sequence $U_{k}(t)$ at the *k*-th antenna branch, the resulting signal becomes

$$U_{k}(t)s(t)=\sum_{l=1}^{N} U_{k}(t)U_{l}(t)v_{l}(t).$$

A common amplitude factor associated with equal signal distribution among the antenna branches is omitted here because it scales the central-frequency power and the total modulation-spectrum power by the same factor and therefore cancels in the efficiency ratio. Both $U_{k}(t)$ and $U_{l}(t)$ have a period $T_{s}$, corresponding to the modulation frequency $f_{s}=1/T_{s}$. As defined in Eq. (S9), their product can be expanded as

$$U_{k}(t)U_{l}(t)=\sum_{m=-\infty}^{+\infty} \alpha_{kl}^{m}e^{j2\pi mf_{s}t}$$

Consequently, the complex envelope produced by the *k*-th branch in the *m*-th harmonic band is $\sum_{l=1}^{N} \alpha_{kl}^{m}v_{l}(t)$ and the corresponding spectral component is centred at $f_{0}+mf_{s}$. To account for the complex excitation coefficients, waveform powers and possible correlations between the synthesized element signals, we define

where denotes the statistical expectation operator. Provided that adjacent harmonic bands are spectrally separated, the total power contained in the *m*-th harmonic band over all antenna branches is

$$P_{m}=\sum_{k=1}^{N} \sum_{l=1}^{N} \sum_{r=1}^{N} R_{lr}\alpha_{kl}^{m}\left(\alpha_{kr}^{m}\right)^{*}$$

This expression incorporates the actual digitally synthesized element waveforms and time sequence interactions, rather than treating each branch as an independently modulated single-tone signal. According to the zero-order coefficients obtained in Eq. (S10), the total useful power at the central frequency is consequently

$$P_{0}=\sum_{k=1}^{N} R_{kk}$$

For the ideal bipolar phase-switching sequences adopted in this work,$|U_{k}(t)|=1.$ Applying the generalized Parseval identity to the Fourier coefficients gives

$$\begin{matrix} \sum_{m=-\infty}^{+\infty} \alpha_{kl}^{m}\left(\alpha_{kr}^{m}\right)^{*}=\frac{1}{T_{s}}\int_{-T_{s}/2}^{T_{s}/2} U_{k}(t)U_{l}(t)\left[U_{k}(t)U_{r}(t)\right]^{*}\text{d}t \\ =\frac{1}{T_{s}}\int_{-T_{s}/2}^{T_{s}/2} U_{l}(t)U_{r}^{*}(t)\text{d}t \\ =\delta_{lr} \end{matrix}$$

The power summed over the complete modulation spectrum is therefore

$$\begin{matrix} P_{\text{all}}=\sum_{m=-\infty}^{+\infty} P_{m} \\ =\sum_{k=1}^{N} \sum_{l=1}^{N} R_{ll} \\ =N\sum_{l=1}^{N} R_{ll} \end{matrix}$$

This power-closure relation shows that ideal bipolar switching does not dissipate power within the modulation model. Instead, it redistributes the power between the desired central-frequency component and the nonzero-order harmonics. The intrinsic central-frequency modulation efficiency is thus defined as

$$\eta_{0}^{\text{th}}=\frac{P_{0}}{P_{\text{all}}}=\frac{1}{N}$$

Accordingly, for the $N=16$ architecture considered in this work,

$$\frac{1}{16}=6.25\% (-12.04\text{ dB})$$

while the remaining 93.75% is redistributed among the nonzero-order harmonics. All nonzero-order harmonic components are included in the denominator and are treated as unused spectral-conversion power in the present system. This analytical result is independent of the particular values of $A_{p}^{k}$,, and the relative waveform powers, provided that the ideal sequence-orthogonality and equal branch-scaling assumptions are satisfied. Equivalently, the theoretical efficiency of 6.25% corresponds to a spectral-conversion loss of approximately 12.04 dB in the useful central-frequency component relative to the total time-modulated RF power. The required central-frequency output power can be restored by introducing corresponding gain in a downstream amplifier after band-pass filtering; however, this amplification does not recover the power redistributed to the rejected harmonics and requires additional DC power consumption. It therefore compensates for the output-power reduction but does not eliminate the intrinsic modulation-efficiency limitation.

This distinction motivates a separate consideration of spectral-conversion efficiency and system-level power efficiency. We stress that $\eta_{0}^{\text{th}}$ is an intrinsic spectral-conversion metric and should not be interpreted as the system power-added efficiency (PAE). Accordingly, under the assumptions adopted above, 6.25% of the temporally modulated RF power is converted into the useful zero-order component, whereas the remaining 93.75% is redistributed among the nonzero-order harmonics and is not utilized in the present implementation. This modulation efficiency is an intrinsic spectral-conversion metric and is distinct from system PAE. System-level PAE additionally depends on the power amplifiers and their operating conditions, as well as feeding, switching, impedance-matching, filtering and antenna losses. The derived modulation efficiency therefore quantifies the architecture-level harmonic-power distribution but does not imply an improvement in system PAE. The present proof-of-concept prototype is intended to validate the proposed radiation-control mechanism and has not been optimized for end-to-end power efficiency.

The proposed 1-bit implementation thus retains the harmonic-power limitation inherent to conventional space-time modulation architectures. Instead, it addresses a different limitation: the restricted radiation-control degrees of freedom imposed by hardware-level switching. Through coherent digital and analog coordination, the proposed framework enables programmable multi-beam waveform synthesis and reduced waveform observability in unintended spatial regions without requiring additional RF chains or higher-resolution phase modulators. Its principal contribution therefore lies in improving the radiation-functionality–hardware-complexity trade-off rather than the energy-efficiency–hardware-complexity trade-off. Improving spectral-conversion efficiency through more efficient temporal modulation or harmonic-reuse strategies remains an important direction for future implementation.

**S1.3 Eavesdropping model, SINR and achievable secrecy rate**

To provide a complementary communication-level interpretation, the following simplified SINR formulation is introduced. Let denote the central-frequency directional response multiplying the *p*-th original baseband signal $s_{p}(t)$. This response is determined by the previously defined amplitude and phase weighting factors $A_{k}^{p}$ and. After band-pass filtering and coherent removal of the common carrier term at $f_{0}$, the equivalent received baseband signal in direction $\theta$ can be expressed as

$$r(\theta,t)=\sum_{p=1}^{Q} F_{p}(\theta)s_{p}(t)+n(\theta,t)$$

where *n(θ,t)* represents the Gaussian white noise. Within this set, *q* denotes the information stream of interest and the subscript $\text{z}$ denotes the masking beam. The average power of $s_{p}(t)$ is defined as

For the legitimate receiver of stream *q*, located in its prescribed direction $\theta_{q}$, the received SINR is

$$\gamma_{\text{L},q}=\frac{P_{q}\left|F_{q}(\theta_{q})\right|^{2}}{\sum_{\begin{matrix} p=1 \\ p\notin q,\text{z} \end{matrix}}^{Q} P_{p}\left|F_{p}(\theta_{q})\right|^{2}+P_{\text{z}}\left|F_{\text{z}}(\theta_{q})\right|^{2}+\sigma_{\text{L}}^{2}}$$

where $\sigma_{\text{L}}^{2}$ denotes the corresponding noise power. The nulling constraints imposed during beam synthesis suppress the other information-bearing components and the masking component at $\theta_{q}$, allowing the intended waveform to be recovered with a high SINR.

For an eavesdropper located at an unintended angle $\theta_{\text{E}}$, the directly observed SINR for stream *q* is

$$\gamma_{\text{E},q}(\theta_{\text{E}})=\frac{P_{q}\left|F_{q}(\theta_{\text{E}})\right|^{2}}{\sum_{\begin{matrix} p=1 \\ p\notin q,\text{z} \end{matrix}}^{Q} P_{p}\left|F_{p}(\theta_{\text{E}})\right|^{2}+P_{\text{z}}\left|F_{\text{z}}(\theta_{\text{E}})\right|^{2}+\sigma_{\text{E}}^{2}}$$

To avoid attributing permanent security benefits to interference from the other information-bearing signals, we further consider a conservative eavesdropper that knows the modulation format and array configuration, is perfectly synchronized, and can ideally identify and cancel all other information streams. The residual worst-case SINR is then

$$\gamma_{\text{E},q}^{\text{wc}}(\theta_{\text{E}})=\frac{P_{q}\left|F_{q}(\theta_{\text{E}})\right|^{2}}{P_{\text{z}}\left|F_{\text{z}}(\theta_{\text{E}})\right|^{2}+\sigma_{\text{E}}^{2}}$$

This expression removes the protection contributed by the other communication streams and retains only the independently generated masking waveform. It therefore provides a more conservative evaluation than direct demodulation of the original composite signal. The masking realization is assumed to be unavailable to the eavesdropper; if it were known exactly and could be perfectly cancelled, no corresponding secrecy advantage would be claimed. Importantly, the eavesdropper SINR is therefore ultimately limited by the co-propagating masking component rather than by receiver noise alone. Blind source-separation or machine-learning methods may still exploit temporal structure, modulation priors, accumulated observations or training data. We do not claim immunity to such processing. Instead, these methods require additional prior knowledge, observation duration and computational resources, which is precisely the interception and exploitation cost that the proposed architecture is intended to increase.

Under the Gaussian-signaling assumption and with the residual interference and receiver noise treated as effective Gaussian noise, the achievable rate of information stream *q* at the legitimate receiver can be expressed as

$$R_{\text{L},q}=\log_{2}\left(1+\gamma_{\text{L},q}\right)$$

Similarly, for an eavesdropper located at $\theta_{\text{E}}$, the achievable rate under the conservative worst-case assumption is

$$R_{\text{E},q}^{\text{wc}}(\theta_{\text{E}})=\log_{2}\left[1+\gamma_{\text{E},q}^{\text{wc}}(\theta_{\text{E}})\right]$$

The difference between $R_{\text{L},q}$ and $R_{\text{E},q}^{\text{wc}}(\theta_{\text{E}})$ represents the channel-rate advantage available to the legitimate receiver. Accordingly, an achievable secrecy rate for stream *q* can be written as

$$R_{s,q}(\theta_{\text{E}})=[\log_{2}\left(1+\gamma_{\text{L,}q}\right)-\log_{2}\left(1+\gamma_{\text{E},q}^{\text{wc}}(\theta_{\text{E}})\right)]^{+}$$

where $[x]^{+}=\max(x,0)$. This expression provides a concise quantitative connection between the synthesized directional responses and the resulting physical-layer secrecy enhancement. We refer to $R_{\text{s},q}$ as an achievable secrecy rate rather than a universal secrecy capacity because it is evaluated for the prescribed waveforms, Gaussian-signaling assumption and specified far-field eavesdropping model, without optimization over all possible input distributions or interception algorithms.

### **SI Section S2 — Optimization formulation and algorithm details**

Under the above signal model and control strategy, the array radiation is governed by the joint action of the digital baseband signals and the applied time-modulation sequence, enabling the assignment of information-bearing and masking functions to different spatial regions. In contrast to conventional time-modulated arrays, where radiation irregularity in unintended directions arises primarily from the time sequence itself, the proposed architecture establishes radiation-domain concealment chiefly through a dedicated masking beam jointly shaped by digital baseband processing and time modulation. For robust spatial waveform concealment, the masking beam is designed to span the entire set of unintended directions, ensuring that the electromagnetic signatures of the information-bearing beams are effectively obscured.

Meanwhile, uniform beam nulling is imposed across all beams along the intended directions to minimize mutual coupling and inter-beam interference, thereby preserving the integrity of the desired waveforms while maintaining independent beam control. Under these constraints, the resulting design problem can be formulated as the following optimization:

$$\text{s.t.}\left\{\begin{matrix} \left|F_{p}(\theta_{l})\right|\leq\varepsilon,\text{ }l\neq p \\ \sum_{k=1}^{N} \left|A_{p}^{k}\right|^{2}\leq1,\text{ }\forall p \end{matrix}\right.$$

where *w_p_* is the corresponding weighting factor for *p*-th beam, and $\Theta$ represents the angular region of interest. *D_p_*(*θ*) denotes the predefined *p*-th target beampattern, expressed as a function of the angle *θ*. In addition, *θ_l_* denotes the *l*-th desired beam scanning direction, and the value of *ɛ* represents the maximum allowable residual magnitude, corresponding to zero depth, which requires suppression. Through this optimization process, $A_{p}^{k}$ and can be obtained.

The overall operational procedure consists of three main stages. First, a time sequence satisfying the constraints in (S7) is generated. Second, the corresponding amplitude and phase weightings required for beam synthesis are obtained using standard array optimization techniques^[1,2]^. Finally, the optimized amplitude weights, phase weights and time sequence are combined to construct the pre-modulated signal *h_p_*(*t*). By loading *h_p_*(*t*) and the time sequence *U_k_*(*t*) into the proposed architecture, the desired set of multiple beams can be realized.

### **SI Section S3 — Simulation configuration and extended numerical and experimental validation**

This section first provides the detailed simulation configuration in the main text. Additional numerical results obtained for a different beam-steering configuration are then presented to demonstrate the robustness of the proposed framework. Finally, both numerical and experimental results using OFDM–QAM (orthogonal frequency division multiplexing – quadrature amplitude modulation) communication waveforms are provided to further verify the waveform versatility and direction-dependent transmission performance of the proposed digitally mediated space–time modulation architecture.

**S3.1 Simulation configuration and signal-processing procedure**

This section provides the detailed simulation configuration supporting the findings reported in the main text. To evaluate waveform observability from different spatial directions, representative time–frequency analysis techniques, including the short-time Fourier transform, Wigner–Ville distribution analysis and cyclostationary spectrum analysis, can be employed^[3]^. In this work, Wigner–Ville distribution analysis, which is particularly sensitive to linear frequency-modulated waveforms, is used to assess the degree of waveform identifiability at unintended observation angles. For the OFDM–QAM communication waveforms, constellation diagrams and error vector magnitude (EVM) are used to evaluate direction-dependent transmission and demodulation performance. The constellation diagrams provide a visual representation of symbol distortion, while EVM quantitatively measures the deviation of the recovered constellation symbols from their ideal reference values.

For comparison, simulations were performed both with and without the masking beam, thereby establishing a baseline case for assessing the effectiveness of spatial waveform concealment. The carrier frequency was set to 1.9 GHz, and the intermediate frequency to 10 MHz. The transmitting aperture was modelled as a 16-element uniform linear array with an interelement spacing of half a wavelength at the carrier frequency. Each element was controlled by a 1-bit phase modulator providing two switching states: 0° and 180°. Each antenna element followed the tightly coupled dipole design^[4]^, with the corresponding active element pattern (AEP) extracted from HFSS and incorporated into the numerical model^[5]^.

Based on the above configuration, the numerical simulations and experimental measurements follow the same signal-processing procedure, as summarized in Supplementary **Fig. S1**. The prescribed beam directions and radiation requirements are first converted into optimized amplitude and phase weights through conventional beam-pattern optimization. These parameters, together with the desired information-bearing signals and masking waveform, are used to construct the digital premodulation mapping and synthesize the composite baseband waveform. Following digital-to-RF conversion, the waveform is synchronously modulated by the time-modulated array to produce the prescribed direction-dependent radiation. The received signals are subsequently processed and evaluated at different observation angles.

**Fig. S1 | Signal-processing flow of the proposed digitally mediated space–time modulation framework.** The desired beam specifications are first converted into optimized amplitude and phase parameters through conventional beam-pattern optimization. These parameters, together with the desired signals and masking waveform, are used to determine the digital premodulation mapping. The resulting composite waveform is then radiated through the space–time-modulated array, followed by direction-dependent reception and signal-performance evaluation.

**Fig. S2 | Optimized time sequence.**

**S3.2 Additional LFMCW beam-steering example**

An additional numerical example is provided to further demonstrate the generality of the proposed framework. In this case, the desired target directions are set to −45°, 10° and 35°. The time-modulation sequence employed is shown in **Fig. S2** and is identical to that used in Fig. 6a of the main text, with only the digital weighting coefficients re-optimized for the new steering configuration. The optimized amplitude and phase weighting coefficients are shown in **Fig. S3**. The corresponding simulation results are presented in **Figs. S4–S8**.

As shown in **Fig. S4**, the introduction of the masking beam does not alter the shapes of the remaining directive beams, indicating negligible inter-beam coupling. **Fig. S5** compares the radiated power distributions with and without the masking beam activated. When the masking beam is present, the signal characteristics in the intended directions remain essentially unchanged, whereas the power distribution in unintended directions becomes noticeably more irregular, which is conducive to reducing waveform identifiability. Furthermore, **Fig. S6 and Fig. S7** present the time–frequency representations of the radiated signals observed at −45°, 10°, 35°, −15°, 20° and 60° without and with the masking beam, respectively. In the absence of masking, the waveform features in unintended directions remain discernible, whereas with the masking beam activated, the time–frequency characteristics in these directions become difficult to identify, while those in the intended directions are preserved.

Whereas the time–frequency distribution describes the temporal evolution of spectral components, the correlation metric provides a direct measure of waveform similarity across spatial locations. The calculated correlation coefficients between the signals observed in the desired directions (−45°, 10° and 35°) and those in unintended directions are summarized in **Fig. S8**. Higher correlation values indicate stronger similarity between the corresponding time–frequency characteristics. As shown in **Fig. S8a**, in the absence of masking radiation, high correlation peaks appear across multiple unintended directions, indicating elevated waveform observability. By contrast, when the masking beam is introduced (**Fig. S8b**), the correlation levels in unintended directions are substantially suppressed, while the waveform characteristics in the desired directions remain essentially unaffected.

These results confirm that the desired directive beams are synthesized with high angular accuracy, while waveform observability in unintended regions is effectively suppressed. Together with the main-text results, this additional example demonstrates the robustness and general applicability of the proposed digitally mediated space–time modulation framework.

**Fig. S3 | Optimized weighting coefficients for beam steering toward −45°, 10°, and 35°.** (**a**) Amplitude; (**b**) phase.

**Fig. S4 | Simultaneous multi-beam radiation enabled by the proposed architecture.** Beam scanning toward −45°, 10°, and 35° without (**a**) and with (**b**) a masking beam.

**Fig. S5 | Radiated power distribution of the proposed architecture.** Beam steering toward −45°, 10°, and 35° without (**a**) and with (**b**) a masking beam.

**Fig. S6 | Time–frequency distributions of signals transmitted by the proposed architecture without a masking beam.** Directions at −45° (**a**), 10° (**b**), 35° (**c**), −15° (**d**), 20° (**e**), and 60° (**f**).

**Fig. S7 | Time–frequency distributions of signals transmitted by the proposed architecture with a masking beam.** Directions at −45° (**a**), 10° (**b**), 35° (**c**), −15° (**d**), 20° (**e**), and 60° (**f**).

**Fig. S8 | Correlation coefficients of the time–frequency distributions for the proposed architecture.** (a) Without a masking beam; (b) with a masking beam.

**S3.3 Numerical and experimental validation using OFDM–QAM communication waveforms**

To further demonstrate the waveform versatility of the proposed digitally mediated space–time modulation framework, an additional communication-waveform simulation was performed using three independent OFDM–QAM signals. The three signals were simultaneously projected toward the prescribed directions of −30°, 0°, and 40°, respectively. The OFDM–QAM signals employ a subcarrier spacing of 2.5 kHz and occupy an overall bandwidth of 400 kHz, matching the bandwidth of the LFMCW waveforms. Apart from the waveform format and its associated parameterization, all other simulation parameters remain identical to those used in the LFMCW radiation case. This example extends the validation beyond the LFMCW sensing waveforms considered in the main text and provides a communication-level assessment of the direction-dependent waveform transmission enabled by the proposed architecture.

Because time–frequency representations are not the most appropriate metric for quantitatively evaluating the demodulation performance and transmission security of digitally modulated communication signals, we adopt the widely used error vector magnitude (EVM) as the principal performance metric. In both simulations and experiments, per-subcarrier complex equalization is applied at the receiver to compensate for linear frequency-selective distortions. The same channel estimation and equalization procedure is applied for all observation angles to ensure a fair and consistent comparison, allowing the evaluation to focus on the proposed architecture’s spatial multiplexing and security performance. To avoid overestimating security, all other information-bearing streams are assumed to be perfectly identified and cancelled during demodulation, so that only receiver noise and the independently generated masking waveform (when enabled) remain as impairments. This conservative treatment removes inter-stream interference as a source of protection and isolates the contribution of the masking waveform.

Without the masking beam, **Fig. S9a** presents the angle–frequency radiation power distribution, while **Fig. S9b** shows the EVM of the three intended signals after demodulation at different observation angles. **Fig. S9c–h** present the corresponding constellation diagrams measured at −30°, 0°, 40°, −15°, 20°, and 60°, respectively. The results show that each OFDM-QAM signal can be correctly demodulated only near its intended direction. At unintended directions, the constellation points become highly dispersed, accompanied by a substantial increase in EVM, indicating severe waveform distortion and unsuccessful demodulation.

We further evaluate the system after introducing the masking beam. **Fig. S10a** shows the resulting angle–frequency radiation power distribution, **Fig. S10b** presents the angular EVM profiles of the three desired signals, and **Fig. S10c–h** show the corresponding constellation diagrams at the same six observation angles. A comparison between **Fig. S9** and **Fig. S10** demonstrates that, although signal demodulation is already difficult at unintended directions without the masking beam, its introduction further increases the EVM and constellation dispersion in these regions, thereby enhancing the direction-dependent waveform obfuscation.

**Fig. S9 | Direction-dependent transmission performance of three OFDM–QAM signals without the masking beam.** (a) Angle–frequency distribution of the normalized radiated power for three independent OFDM–QAM signals directed toward −30°, 0°, and 40°, respectively. (b) EVM of the three signals as a function of observation angle. (c–h) Demodulated constellation diagrams observed at −30°, 0°, 40°, −15°, 20°, and 60°, respectively.

**Fig. S10 | Direction-dependent transmission performance of three OFDM–QAM signals with the masking beam.** (a) Angle–frequency distribution of the normalized radiated power for three independent OFDM–QAM signals directed toward −30°, 0°, and 40°, respectively, in the presence of the masking beam. (b) Corresponding EVM of the three signals as a function of observation angle. (c–h) Demodulated constellation diagrams observed at −30°, 0°, 40°, −15°, 20°, and 60°, respectively.

The simulation results are further validated experimentally in **Fig. S11** and **Fig. S12**. Without the masking beam, **Fig. S11a** presents the measured angle–frequency distribution of the normalized received power, while **Fig. S11b** shows the measured angular EVM profiles of the three OFDM–QAM signals. **Fig. S11c–h** present the corresponding measured constellation diagrams at −30°, 0°, 40°, −15°, 20°, and 60°, respectively. Consistent with the simulations in **Fig. S9**, the constellation points remain clearly clustered near the intended directions but become substantially dispersed in the unintended directions, with a corresponding increase in EVM. These results experimentally confirm the direction-dependent transmission and demodulation characteristics of the three parallel spatial data streams.

**Fig. S12** presents the corresponding experimental results with the masking beam. The measured angle–frequency power distribution in **Fig. S12a**, angular EVM profiles in **Fig. S12b**, and constellation diagrams in **Fig. S12c–h** consistently show that the intended signals remain recoverable in their prescribed directions, whereas their observability and demodulation quality are further degraded in the unintended directions. Compared with **Fig. S11**, the increased EVM and constellation dispersion in **Fig. S12** experimentally verify the additional waveform-obfuscation effect introduced by the masking beam.

Overall, the experimental results in **Fig. S11** and **Fig. S12** agree well with the corresponding simulations in **Fig. S9** and **Fig. S10** in terms of the intended beam directions, angular EVM trends, and direction-dependent constellation dispersion. A narrowband spectral component is observed at the center frequency in the measured angle–frequency distributions. This component is likely attributable to residual carrier or local-oscillator leakage arising from imperfect isolation or DC offsets in the experimental RF chain. Because it is confined to a narrow frequency region, it does not materially affect the measured angular EVM trends, constellation behavior, or the overall conclusions of the experiment.

**Fig. S11 | Experimental validation of the direction-dependent transmission performance of three OFDM–QAM signals without the masking beam.** (a) Measured angle–frequency distribution of the normalized radiated power for three independent OFDM–QAM signals transmitted toward −30°, 0°, and 40°, respectively. (b) Measured EVM of the three signals as a function of observation angle. (c–h) Demodulated constellation diagrams measured at −30°, 0°, 40°, −15°, 20°, and 60°, respectively.

**Fig. S12 | Experimental validation of the direction-dependent transmission performance of three OFDM–QAM signals with the masking beam.** (a) Measured angle–frequency distribution of the normalized radiated power for three independent OFDM–QAM signals transmitted toward −30°, 0°, and 40°, respectively, in the presence of the masking beam. (b) Corresponding measured EVM of the three signals as a function of observation angle. (c–h) Demodulated constellation diagrams measured at −30°, 0°, 40°, −15°, 20°, and 60°, respectively.

### **SI Section S4 — Experimental calibration and measurement procedures**

In the experiment, the core phase modulation switch is constructed by two chips (BW1257), which are designed and assembled by Hebei Semiconductor Research Institute. The adopted phase switch can generate time-modulated switching between 0° and 180° phase weighting. The measured transmission phases corresponding to the two operational states, as well as the phase difference between these states, are shown in **Fig. S13a** and **Fig. S13b**, respectively. It can be found that the phase difference between the two states is nearly 180°. During the measurement, the carrier frequency is set to 1.9 GHz, and the corresponding modulation phase difference is approximately 180°.

**Fig. S14** depicts the diagram of experimental setup. As shown in **Fig. S14**, the experimental setup included an oscilloscope (Siglent SDS812X), a spectrum analyzer (Rohde & Schwarz FPC 1500), a local oscillator (LO) signal generator (Agilent N9310A), and a field-programmable gate array (FPGA, Xilinx AX7103), a digital to analog converter (DAC, Analog Devices AD9767), low noise amplifier (LNA, Agilent 8449B), and cables linking these devices, etc. As illustrated in **Fig. S14**, during the experiment, the FPGA simultaneously controls the DAC to generate baseband signals, which are then up-converted by a frequency converter and distributed through a power divider to each RF switch. The RF switches are controlled by the time sequence generated synchronously by the FPGA to modulate the input signals. Since both the baseband signal generation and the time sequence generation share the same FPGA clock, they are naturally synchronized. This synchronization is critical to preserve the deterministic timing relationships necessary for coordination between the digital and analog domains. The common-clock implementation establishes a fixed relative delay between baseband generation and time modulation. Reproducing the proposed digital and analog coupling therefore requires synchronized generation of the baseband signals and time sequences. Subsequently, each antenna element radiates the modulated signals into space, where they are received by a horn antenna. The received signals are amplified by an LNA and then split into two paths: one path is fed into a spectrum analyzer to measure the frequency-domain characteristics, while the other path is down-converted by a mixer and sent to an oscilloscope to capture the time-domain signals. Finally, the data from both the spectrum analyzer and the oscilloscope are transferred to a PC for comprehensive performance analysis for the proposed architecture.

Based on this experimental configuration, the signal generation and radiation mechanism can be further explained as follows. The waveforms assigned to different beams are first combined in the digital baseband. The subsequent time-modulation stage introduces a convolution operation in the frequency domain, which effectively shifts, weights and superposes the spectral components of the constituent signals, resulting in a composite radiated waveform centred at a common carrier frequency.

As a consequence, unlike conventional space–time modulation architectures, the proposed system supports simultaneous multi-beam radiation while maintaining a unified centre frequency. To facilitate independent measurement of the radiation patterns associated with each beam, a small and distinct frequency offset is deliberately applied to each waveform in the digital baseband. This spectral tagging allows the individual beams to be unambiguously distinguished at the receiver using a spectrum analyser.

**Fig. S13 | Measured phase-switch parameters.** (**a**) Transmission phases for the two operational states; (**b**) phase difference between the two states.

**Fig. S14 | Experimental configuration.**

### **SI Section S5 — Extended discussion on scalability and limitations**

**S5.1 Scalability with array size**

The proposed digitally mediated space–time modulation framework exhibits favourable scalability with increasing array size. As the number of antenna elements grows, the radiation-control degrees of freedom introduced at the digital baseband expand proportionally, enabling a corresponding increase in the number of independently controllable directive and masking beams. This scaling behaviour allows the framework to accommodate more complex radiation patterns and multi-beam configurations without requiring a proportional increase in time-modulation hardware complexity.

The computational cost associated with beam synthesis and optimization increases polynomially with array dimension, dominated by the evaluation of array responses over sampled angular grids. Importantly, this computational growth remains tractable for large-scale arrays and is well suited for parallel implementation on modern computing platforms, preserving the practical feasibility of the proposed approach as system dimensions expand.

**S5.2 Scalability under DAC constraints**

In principle, the space–time modulation mechanism does not impose a fixed upper bound on the number of independently synthesized beams, provided that sufficient spatial degrees of freedom and digital-processing resources are available. In practical implementations, however, the achievable beam number is governed by a coupled beam–bandwidth–precision trade-off rather than by the available digital baseband bandwidth alone. Because the transmission and masking waveforms are digitally combined and generated through a shared DAC, the digital processing chain must accommodate the occupied bandwidth, dynamic range and real-time synthesis workload of the resulting composite waveform. When multiple beams carry cochannel signals, increasing the beam number does not necessarily broaden the occupied bandwidth, because the constituent waveforms overlap spectrally; instead, it mainly increases the computational workload, composite-waveform peak-to-average power ratio and required effective converter resolution. By contrast, when different beams occupy separate frequency channels, the total occupied bandwidth spans all allocated subbands and therefore increases with both the number and bandwidth of the signals. Moreover, the occupied bandwidth determines the minimum time-modulation frequency required to separate the desired central-frequency component from adjacent modulation harmonics, which in turn sets the sequence-chip rate and DAC sampling-rate requirement. The practical scalability of the architecture must therefore be assessed jointly in terms of occupied bandwidth, modulation frequency, sequence length, DAC sampling rate and effective resolution, as quantified below.

Let $B_{\text{occ}}$ denote the total occupied bandwidth of the composite transmission and masking waveforms, $f_{\text{IF}}$ the intermediate frequency, $f_{s}$ the fundamental time-modulation frequency, and *M* the number of sequence time slots within one modulation period. To avoid overlap between the desired zero-order band and its adjacent modulation harmonics, the modulation frequency should approximately satisfy

$$f_{s}\geq B_{\text{occ}}+B_{\text{tr}}$$

where *B_tr_* accounts for the required filter transition band. The corresponding sequence-chip rate is

$$R_{\text{chip}}=Mf_{s}$$

For the real-valued IF implementation used in this work, the DAC sampling rate must therefore satisfy both the IF Nyquist condition and the sequence-update requirement:

$$F_{\text{DAC}}\geq\max\left[2\left(f_{\text{IF}}+\frac{B_{\text{occ}}}{2}+B_{\text{g}}\right),LMf_{s}\right]$$

where $B_{\text{g}}$ is an implementation guard band and $L\geq1$ is the number of DAC samples used per sequence time slot. Because *N* mutually orthogonal sequences require a sequence dimension of at least $M\geq N$, an optimistic lower-order scaling is

$$F_{\text{DAC}}\gtrsim LN\left(B_{\text{occ}}+B_{\text{tr}}\right)$$

For example, even with the optimistic choices (*M*=*N*=16), (*L*=1), and negligible transition bandwidth, a 100 MHz occupied bandwidth requires a sequence-update rate of at least 1.6 GSPS; using two samples per time slot increases this value to 3.2 GSPS. For a 1 GHz signal bandwidth, the corresponding values become 16 and 32 GSPS, respectively. Longer sequences, additional oversampling and practical filter-transition requirements further increase these rates. These estimates confirm that extending the present single-DAC architecture to hundreds-of-megahertz or gigahertz bandwidths would impose a severe sampling-rate and digital-power burden.

The number of beams does not, however, necessarily multiply the required DAC bandwidth. When the transmission and masking waveforms are transmitted over the same spectral band, their digital superposition retains approximately the same occupied bandwidth; their number instead increases the waveform-generation workload, peak-to-average power ratio and dynamic-range requirement. If the signals occupy distinct frequency channels, $B_{\text{occ}}$ becomes the span of all occupied subbands and consequently increases with the number of channels.

The DAC resolution can be estimated from the power fraction of the weakest constituent waveform in the composite DAC signal. Let $\Gamma$ denote the composite waveform peak-to-average power ratio, $\beta_{i}=P_{i}/P_{\text{tot}}$ the power fraction of the *i*-th transmission waveform, and $L_{\text{imp}}$ an implementation margin accounting for nonidealities. Following the pseudo-quantization-noise model for a practical converter with finite ENOB and input power back-off^[6]^, the signal-to-quantization noise ratio (SQNR) associated with the *i*-th waveform can be approximated as

$$\text{SQNR}i\approx6.02b_{\text{eff}}+4.77-\Gamma_{\text{dB}}+10\log_{10}\beta_{i}-L_{\text{imp}}$$

where $b_{\text{eff}}$ is the effective number of bits (ENOB) rather than the nominal DAC resolution. For *Q* equal-power desired waveforms and a masking waveform whose power is $\rho$ times that of one transmission waveform, $\beta_{i}=1/(Q+\rho)$, giving

$$b_{\text{eff}}\gtrsim\frac{\text{SNR}_{\text{req}}+\Gamma_{\text{dB}}+10\log_{10}(Q+\rho)+L_{\text{imp}}-4.77}{6.02}$$

As an illustrative wideband case, four equal-power transmission waveforms, a masking waveform having the same power as their combined power $(\rho=4)$, a 10 dB composite PAPR, a 30 dB per-waveform SQNR requirement and a 3 dB implementation margin require approximately 8 effective bits. Because the ENOB of a multi-giga sample-per-second DAC is generally lower than its nominal resolution and is further limited by clock jitter, nonlinear distortion and spurious-free dynamic range, a higher nominal bit depth would be required in practice. Stronger masking, additional beams or more stringent EVM requirements would further increase this requirement.

The millimeter-wave carrier frequency does not by itself determine the DAC sampling rate in an architecture employing analog frequency upconversion; the dominant quantities are the instantaneous signal bandwidth, IF, and sequence-chip rate. In practice, state-of-the-art high-speed DACs already achieve sampling rates in the range of tens of GSPS with effective resolutions of approximately 6–10 bits, which are sufficient for many wideband communication and radar waveform generation scenarios. Direct millimeter-wave synthesis by the DAC would, however, still impose substantially more stringent sampling-rate and clock-jitter requirements than those typically supported by current commercial devices, making analog upconversion a more practical choice for system implementation.

The present 10 MHz IF experiment provides a proof-of-concept validation of the proposed digitally mediated radiation-control mechanism. More importantly, the quantitative analysis above, together with a comparison against representative commercial RF DACs, demonstrates that practical implementation is feasible within realistic combinations of signal bandwidth, beam number and converter resolution. For example, the estimated sampling rate of 1.6–3.2  GSPS required for a 100 MHz occupied bandwidth is well below the maximum sampling rate of 20  GSPS provided by the typical commercial high-speed DAC^[7]^. Assuming the same sampling-rate-to-bandwidth ratio, such a device could theoretically support occupied signal bandwidths of approximately 0.625–1.25 GHz, with a maximum value of about 1.25 GHz, subject to practical analog-bandwidth, effective-resolution and data-throughput constraints. The analysis also delineates the practical operating boundary of a single-DAC implementation: as the occupied bandwidth approaches the gigahertz range, the simultaneous requirements on sampling rate, effective resolution and digital-processing power become increasingly stringent. Beyond this regime, these constraints can be further alleviated through parallel or time-interleaved DACs, subarray partitioning, reduced-length orthogonal sequence design, mixed-signal channelization and partial analog beamforming. These strategies distribute the sampling and processing workload across multiple hardware resources and therefore provide practical routes toward wider bandwidths and larger antenna apertures, although at the cost of increased system complexity and power consumption. Overall, the proposed architecture is established as an experimentally validated and analytically supported engineering framework. The analysis above delineates the operating limits of the current single-DAC implementation and identifies feasible hardware strategies for extending its supported bandwidth and scalability.

**S5.3 Hardware constraints and implementation limits**

Practical hardware non-idealities inevitably introduce deviations from the idealized signal model. Non-ideal switching behaviour, timing jitter and amplifier nonlinearities may lead to reduced null depths, minor beam distortions and additional spectral artifacts, as partially reflected in the experimental measurements. Nevertheless, these effects do not compromise the fundamental radiation-control mechanisms enabled by digitally mediated space–time modulation. The core capabilities of simultaneous multi-beam waveform synthesis and spatially selective waveform concealment remain robust under such imperfections.

**S5.4 Masking effectiveness and adversarial knowledge assumptions**

The results presented in this work assume no prior knowledge of unintended receiver locations. Under this assumption, the masking beam is distributed broadly across unintended spatial regions to suppress waveform observability in a spatially uniform manner. When partial spatial information becomes available, the same digitally mediated control framework can be adapted to concentrate masking radiation toward specific angular sectors, enabling more targeted suppression of waveform observability and further enhancing radiation-domain physical-layer security.

**S5.5 Implementation trade-offs, limitations and future directions**

The proposed framework involves two principal implementation trade-offs. First, it requires greater digital-processing complexity than conventional space–time modulation because multiple desired waveforms and the masking waveform must be digitally premodulated and combined into a composite baseband signal. The resulting computational load, memory usage and digital data throughput increase with the number and bandwidth of the synthesized waveforms. In return, part of the radiation-control complexity is transferred from the RF hardware to the digital domain, enabling enhanced spatial and waveform control without introducing additional RF chains or increasing the resolution of the 1-bit time modulators. In the present proof-of-concept system, the premodulation parameters and composite waveforms are calculated in advance, thereby keeping the real-time processing burden manageable. Second, deterministic synchronization between the digitally premodulated waveforms and the analog time-modulation sequences is essential. A relative timing mismatch alters their coherent interaction and can degrade the intended spatial waveform synthesis. In the current experiment, a common-clock FPGA coordinates both the DAC output and the switching signals applied to the time modulators, establishing a fixed timing relationship between the digital and analog modulation domains and avoiding synchronization drift associated with independent clocks. Scaling the framework to larger or distributed arrays will require more accurate clock distribution, delay calibration and drift compensation.

Several limitations of the current implementation warrant further investigation. Extremely wideband waveforms may require higher time-modulation rates to preserve waveform fidelity, which could impose additional demands on switching hardware as well as digital sampling rate and data throughput. In addition, highly dynamic multipath environments may introduce coupling effects that are not explicitly captured in the present model and could influence masking effectiveness. Addressing these challenges and extending the framework to distributed or reconfigurable array architectures represent promising directions for future research. Overall, the proposed method trades increased digital-processing and synchronization requirements for expanded radiation-control flexibility while retaining a simple 1-bit time-modulated RF architecture.

**References**

1. Yan, K. & Lu, Y. Side lobe reduction in array-pattern synthesis using genetic algorithm. *IEEE Trans. Antennas Propag.* **45**, 1117–1122 (1997).
2. Nagar, J. & Werner, D. H. Multiobjective optimization for electromagnetics and optics: an introduction and tutorial based on real-world applications. *IEEE Antennas Propag. Mag.* **60**, 58–71 (2018).
3. Pace, P. E. Detecting and Classifying Low Probability of Intercept Radar (Artech House, 2009).
4. Zhou, L., Yang, S., Guo, Z. & Chen, Y. A wideband dual-polarized dipole linear array with resistive loading. In *Proc. IEEE MTT-S Int. Microw. Workshop Ser. Adv. Mater. Process. RF THz Appl.* 1–3 (2016).
5. Pozar, D. M. The active element pattern. *IEEE Trans. Antennas Propag.* **42**, 1176–1178 (1994).
6. Riedel, I., Windisch, M. & Fettweis, G. On the impact of analog-to-digital conversion on blind I/Q imbalance parameter estimation in low-IF receivers. In *Proc. 2005 International Conference on Wireless Networks, Communications and Mobile Computing* (IEEE, 2005).
7. Texas Instruments. DAC39RFx10-SP and DAC39RFx10-SEP: 10.4 or 20.8 GSPS, 16-bit, dual- and single-channel, multi-Nyquist digital-to-analog converters with JESD204C interface. Data Sheet, Rev. A (2026).
